# Dysfunction of motor cortices in Parkinson’s disease

**DOI:** 10.1093/cercor/bhae294

**Published:** 2024-07-26

**Authors:** Hong-Yuan Chu, Yoland Smith, William W Lytton, Scott Grafton, Rosa Villalba, Gunasingh Masilamoni, Thomas Wichmann

**Affiliations:** Aligning Science Across Parkinson’s (ASAP) Collaborative Research Network, Chevy Chase, MD 20815, United States; Department of Pharmacology and Physiology, Georgetown University Medical Center, 3900 Reservoir Rd N.W., Washington D.C. 20007, United States; Aligning Science Across Parkinson’s (ASAP) Collaborative Research Network, Chevy Chase, MD 20815, United States; Department of Neurology, School of Medicine, Emory University, 12 Executive Drive N.E., Atlanta, GA 30329, United States; Emory National Primate Research Center, 954 Gatewood Road N.E., Emory University, Atlanta, GA 30329, United States; Aligning Science Across Parkinson’s (ASAP) Collaborative Research Network, Chevy Chase, MD 20815, United States; Department of Physiology & Pharmacology, SUNY Downstate Medical Center, 450 Clarkson Avenue, Brooklyn, NY 11203, United States; Department of Neurology, Kings County Hospital, 451 Clarkson Avenue,Brooklyn, NY 11203, United States; Aligning Science Across Parkinson’s (ASAP) Collaborative Research Network, Chevy Chase, MD 20815, United States; Department of Psychological and Brain Sciences, University of California, 551 UCEN Road, Santa Barbara, CA 93106, United States; Aligning Science Across Parkinson’s (ASAP) Collaborative Research Network, Chevy Chase, MD 20815, United States; Emory National Primate Research Center, 954 Gatewood Road N.E., Emory University, Atlanta, GA 30329, United States; Aligning Science Across Parkinson’s (ASAP) Collaborative Research Network, Chevy Chase, MD 20815, United States; Emory National Primate Research Center, 954 Gatewood Road N.E., Emory University, Atlanta, GA 30329, United States; Aligning Science Across Parkinson’s (ASAP) Collaborative Research Network, Chevy Chase, MD 20815, United States; Department of Neurology, School of Medicine, Emory University, 12 Executive Drive N.E., Atlanta, GA 30329, United States; Emory National Primate Research Center, 954 Gatewood Road N.E., Emory University, Atlanta, GA 30329, United States

**Keywords:** Parkinson’s disease, cerebral cortex, basal ganglia, dopamine, pathophysiology

## Abstract

The cerebral cortex has long been thought to be involved in the pathophysiology of motor symptoms of Parkinson’s disease. The impaired cortical function is believed to be a direct and immediate effect of pathologically patterned basal ganglia output, mediated to the cerebral cortex by way of the ventral motor thalamus. However, recent studies in humans with Parkinson’s disease and in animal models of the disease have provided strong evidence suggesting that the involvement of the cerebral cortex is much broader than merely serving as a passive conduit for subcortical disturbances. In the present review, we discuss Parkinson’s disease–related changes in frontal cortical motor regions, focusing on neuropathology, plasticity, changes in neurotransmission, and altered network interactions. We will also examine recent studies exploring the cortical circuits as potential targets for neuromodulation to treat Parkinson’s disease.

## Introduction

The motor signs and symptoms of Parkinson’s disease (PD) are in large part a consequence of the degeneration of dopaminergic neurons in the substantia nigra pars compacta (SNc) and the resulting loss of dopamine (DA) in the basal ganglia. Most studies of the pathophysiologic roots of parkinsonism have focused on the effects of DA loss on basal ganglia activities, demonstrating that it is associated with a greater tendency of basal ganglia neurons to fire in synchronized oscillatory and nonoscillatory bursts and that it leads to numerous changes of dendritic and synaptic morphology (Bergman et al. 1998; Day et al. 2006; Hammond et al. 2007; Galvan and Wichmann 2008; Mallet et al. 2008; Fieblinger et al. 2014; Mathai et al. 2015; Parker et al. 2016; Chu et al. 2017; Wichmann 2018; Willard et al. 2019).

Traditional models of cortico-basal ganglia–thalamocortical network interactions (Albin et al. 1989; DeLong 1990) posit that the subcortical DA loss eventually leads to disturbances of processing in the principal cortical targets of outflow from motor portions of the basal ganglia, i.e. the supplementary motor area (SMA) and the primary motor cortex (M1). However, exciting new evidence suggests that the cortical involvement is not only that of an otherwise healthy passive conduit of subcortical disturbances (as predicted by traditional models) but also that the motor cortices are sites of independent local anatomical and functional abnormalities that contribute to motor dysfunctions seen in PD. In this review, we will discuss the anatomical, electrophysiological, and human imaging studies that have assessed PD-related changes in frontal cortical motor regions and critically examine recent studies exploring the cortical circuits as potential targets for neuromodulation to treat PD.

## Anatomy of frontal motor cortices

**Fig. 1:**
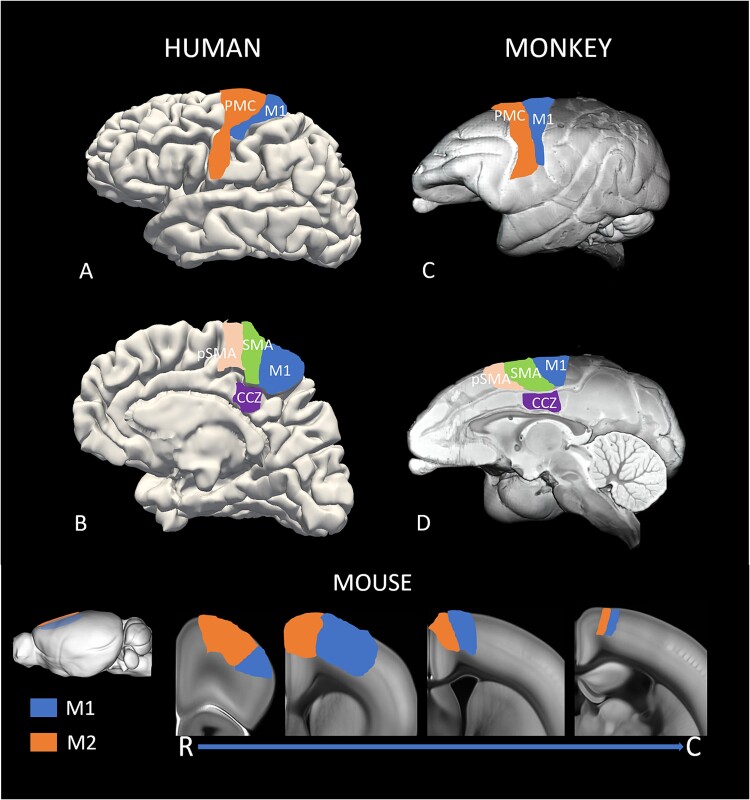
Regional maps of the localization of motor cortices. A–D) The lateral and medial surface of the human and macaque brains. Bottom) The localization of M1 and M2 cortices on the lateral surface and in coronal sections through the rostrocaudal axis of the mouse brain. The inset shows the abbreviations: CCZ: caudal cingulate zone, M1: primary motor cortex, M2: secondary motor cortex, PMC: pre-motor cortex, pSMA: presupplementary motor area, SMA: supplementary motor area. The lateral and sagittal views of the macaque and mouse brains are from: Scalable Brain Atlas - Neuroanatomy at your fingertips (incf.org). The coronal sections of the mouse brain are from the Allen Brain Atlas website.

The human and monkey frontal motor cortices include several regions that are heavily connected with the basal ganglia and contribute to the corticospinal system. At least five motor cortical regions have been identified in the primate frontal lobe: the primary motor cortex (also called M1 or area 4), the premotor cortex (PMC) that lies along the lateral surface of the hemisphere, as well as the pre-SMA, the SMA, and the caudal cingulate zone (CCZ) on the medial wall of the hemisphere (Dum and Strick 2002; Grafton and Volz 2019; Strick et al. 2021) (Fig. 1). All of these areas receive significant inputs from basal ganglia–receiving regions of the motor thalamus. In rodents, the motor cortex is divided into two major subregions called M1 and M2 (Fig. 1). Although much remains to be known about the homology between these areas and the different primate motor cortices, the rodent M2 is often seen as the homologue of the primate PMC/SMA (Strick et al. 2021).

## Cortical neurochemical changes associated with parkinsonism

### Neuromodulators

#### Catecholamines

There is compelling evidence that the norepinephrine (NE) supply to the motor cortices is severely damaged in PD, even before motor symptoms develop (Chaudhuri et al. 2023). Thus, positron emission tomography (PET) imaging in patients with PD showed decreased binding of DA or NE markers in M1 (Brooks and Piccini 2006; Moriguchi et al. 2017; Sommerauer et al. 2018; Andersen et al. 2020). Postmortem immunohistochemical and biochemical studies also demonstrated a significant decrease in NE and a more modest reduction in dopaminergic innervation of M1 and other motor cortices in PD patients (Scatton et al. 1983; Gaspar et al. 1991; Buddhala et al. 2015; Chaudhuri et al. 2023).

**Fig. 2:**
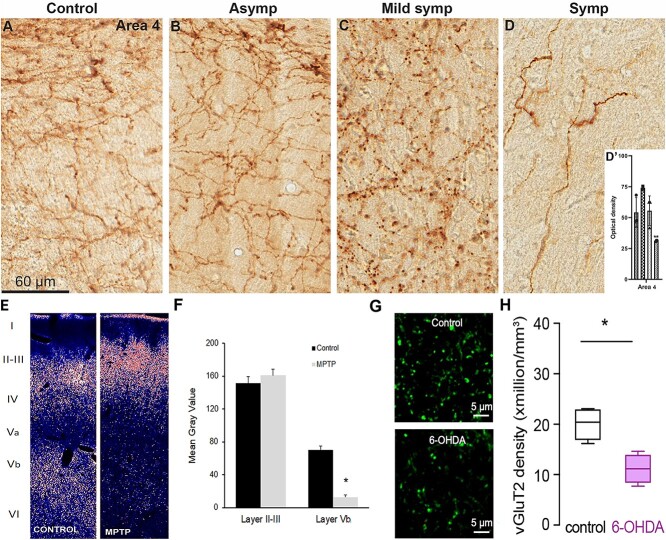
Changes in cortical dopaminergic and glutamatergic innervation patterns in parkinsonian monkeys. A–D) TH-immunostained axonal profiles in the M1 of a control monkey (A) compared with a motor asymptomatic (B), mildly symptomatic (C), and symptomatic (D) MPTP-treated rhesus monkey. D’) Average optical density measurements of TH immunostaining in M1 in the 4 groups of monkeys used in this study. From left to right, the bars represent values from each animal in the order depicted in A–D) (see Masilamoni et al. 2022 for more details). E, F) vGluT2-immunostained profiles in M1 of a control and an MPTP-treated parkinsonian monkey (E), along with the corresponding optical density measurements in layer II–III and Vb (F). Note the significant reduction in vGluT2 labeling in layer Vb of the MPTP-treated monkey (see Villalba et al. 2021 for more details). G, H) Confocal images of vGluT2-immunolabled terminal profiles in layer V of M1 from a control and a 6-OHDA-treated mouse (G) and summarized results (see Chen et al. 2023 for details). A–F) are from Villalba et al. 2021, while F) and G) are from Chen et al. 2023. Both parts of this figure are used with permission.

Studies of the cortical catecholaminergic innervation in 1-methyl-4-phenyl-1,2,3,6-tetrahydropyridine (MPTP)-treated moderately parkinsonian monkeys showed that more than 70% of tyrosine hydroxylase (TH) immunostaining (Fig. 2A–D), indicative of the DA or NE innervation, is lost in M1 (Jan et al. 2003; Masilamoni et al. 2022). Studies of the DA and NE tissue concentrations in several frontal cortical regions of such animals also showed over 50% loss of both catecholamines in PMC, M1, and SMA (Elsworth et al. 1990; Schneider and Kovelowski 1990; Pifl et al. 1991). Interestingly, these changes were also found in motor-asymptomatic MPTP-treated monkeys with partial nigrostriatal dopamine loss, suggesting that the cortical catecholaminergic denervation begins early during the development of parkinsonism (Pifl et al. 1991). Although the SMA also underwent DA and NE loss in these monkeys, changes were not as pronounced as in the M1 and PMC, reaching significance only in symptomatic animals (Pifl et al. 1991).

In recent years, it has become clear that the DA innervation plays an important role in M1 where DA release may regulate the excitability, synaptic plasticity, and spine turnover of cortical pyramidal neurons and may contribute to aspects of motor learning (Luft and Schwarz 2009; Molina-Luna et al. 2009; Hosp et al. 2011a; Hosp et al. 2011b; Hosp and Luft 2013; Rioult-Pedotti et al. 2015; Cousineau et al. 2022; Plateau et al. 2023). In rodents, most of the DA innervation of M1 originates from the ventral tegmental area (VTA), whereas the primate M1 receives its DA inputs mostly from the dorsal tier of the substantia nigra pars compacta (SNc) (Williams and Goldman-Rakic 1998; Molina-Luna et al. 2009; Hosp et al. 2011b). In rodents, the dopaminergic mesocortical innervation and the mesostriatal projection largely originate from different populations of VTA neurons (Beier et al. 2015). This question is less studied in NHPs (see Gaspar et al. 1992). The dopaminergic innervation of M1 is far more substantial in nonhuman primates (NHPs) and humans than it is in rodents (Lewis et al. 1987; Berger et al. 1988; Gaspar et al. 1989; Berger et al. 1991; Gaspar et al. 1991; Gaspar et al. 1992; Williams and Goldman-Rakic 1993, 1998; Lewis and Gonzalez-Burgos 2006). In fact, M1 is the most densely DA-innervated cortical region in the primate brain (Gaspar et al. 1989; Berger et al. 1991).

Receptors belonging to the D1- and D2-receptor families (henceforth called D1- and D2-receptors, respectively) are expressed in pyramidal neurons and subsets of GABAergic interneurons in M1 across species. Blockade of either receptor subtype impairs motor learning in rodents (Camps et al. 1990; Lidow et al. 1991; Gaspar et al. 1995; Lidow 1995; Luft and Schwarz 2009; Molina-Luna et al. 2009; Cousineau et al. 2020; Cousineau et al. 2022). Although there is evidence that D1 and D2 receptors modulate the excitability of pyramidal neurons in the rodent M1, the literature on this topic remains highly discrepant, mainly due to the use of different preparations and the heterogeneity of M1 pyramidal neurons (see Luft and Schwarz 2009; Cousineau et al. 2022; Plateau et al. 2023 for details). Both DA receptor subtypes regulate glutamatergic and GABAergic transmission in the rodent M1 (Cousineau et al. 2020; Cousineau et al. 2022) and may modulate the turnover rate of dendritic spines on pyramidal neurons (Guo et al. 2015) (see below).

Selective ablation/modulation of the mesocortical DA system to the rodent M1 (Luft and Schwarz 2009; Molina-Luna et al. 2009; Hosp et al*.* 2011a; Hosp et al*.* 2011b; Hosp and Luft 2013; Rioult-Pedotti et al. 2015) disrupts the acquisition, but not the maintenance, of newly learned motor skills, raising the possibility that deficits in motor learning seen in the DA-depleted state may originate in part from the loss of DA in M1.

D1 and D2 dopamine receptors are also expressed in several motor cortices (M1, SMA, pre-SMA, PMC, CCz) in primates (Richfield et al. 1989; Lidow et al. 1991), but their role in synaptic regulation remains poorly understood. Given the fact that DA inputs to M1 are far stronger in primates than in rodents, future NHP studies to examine the role of M1 DA in motor processing, and the potential contribution of changes in cortical DA transmission to the development of abnormal neuronal activity seen in NHP models of parkinsonism must be conducted.

Overall, there is compelling evidence that the NE and DA innervation of motor cortices is diminished in PD, even at early stages of the disease, but the contribution of these changes to parkinsonian motor signs remains poorly understood. Further investigations of the impact of these changes on motor cortical functions are warranted.

#### Serotonin

There is significant neuropathological and biochemical evidence that the ascending serotonergic system from the dorsal and median raphe to the cerebral cortex also undergoes neurodegeneration in PD, with a consensus that the pathology in this system may predominantly affect prefrontal cortical areas and contribute mainly to PD-related neuropsychiatric, cognitive, and sleep complaints (Scatton et al. 1983; Ogawa et al. 1992; Kish 2003; Guttman et al. 2007; Albin et al. 2008; Azmitia and Nixon 2008; Politis et al. 2010; Politis et al. 2012; Buddhala et al. 2015; Maillet et al. 2016; Blesa et al. 2022). Information about the state of the serotonergic innervation of motor cortices in PD is limited.

The detected pathology of the motor cortical serotonergic innervation in MPTP-treated NHPs ranges from reports of a significant loss of serotonin in M1, PMC, and SMA in asymptomatic or symptomatic NHPs (Pifl et al. 1991; Perez-Otano et al. 1994; Kanazawa et al. 2017) to reports of no change of the cortical serotoninergic innervation (Beaudoin-Gobert et al. 2015; Engeln et al. 2015; Ballanger et al. 2016). At least in part, technical differences, such as the use of different MPTP dosing schedules, survival times after the MPTP treatment, and methods used to detect changes of the serotonergic innervation (biochemistry, immunostaining, PET imaging) may account for these discrepancies.

In a recent study, a significant reduction of the serotonergic innervation of M1 and SMA was reported in motor-asymptomatic MPTP-treated NHPs (Masilamoni et al. 2022), along with a significant loss of serotonergic raphe neurons in chronically MPTP-treated parkinsonian NHP, consistent with human neuropathology and PET imaging data (Halliday et al. 1990a; Halliday et al. 1990b; Politis et al. 2012) and evidence from resting-state functional MRI (rsfMRI) data showing reduced connectivity between the raphe nuclei and several frontal, temporal, occipital, and limbic cortical regions in patients with advanced PD (Wang et al. 2023).

Taken together, very little is known about the state of serotonergic innervation of motor cortices in early- and late-stage parkinsonian patients and related animal models. Furthermore, the available results are highly variable and discrepant. Additional studies are warranted to assess the potential contribution of serotonergic denervation of motor cortices to the development of parkinsonism.

#### Acetylcholine

PD is associated with a loss of basal forebrain corticopetal cholinergic (BFCC) neurons (Candy et al. 1986; Liu et al. 2015; Albin et al. 2022), leading to reduced cortical cholinergic innervation, with the extent of loss correlating with the magnitude of cognitive deficits in in these patients (Candy et al. 1986; Liu et al. 2015). Imaging studies of regional differences of the density of cholinergic terminals across cortical and subcortical regions have strengthened this view and suggested that BFCC dysfunction may contribute to gait disorders in human PD (Pasquini et al. 2021; Albin et al. 2022; Bohnen et al. 2023; Okkels et al. 2023). Together, the results of these studies concur that high-order parietal and temporal areas are the main cortical regions affected by the degeneration of the BFCC system and that the extent of cholinergic denervation of these areas is positively correlated with the severity of late cognitive impairments, dementia, and gait/balance-related deficits in PD (Albin et al. 2022). Although little is known about the consequence of BFCC cell loss on the cholinergic innervation of motor cortices, a recent PET imaging study showed a positive correlation between the reduction of [^18^F]FEOBV uptake, a specific marker of the vesicular acetylcholine transporter, in the pre- and postcentral gyri of nondemented early PD patients and the severity of motor symptoms in their extremities (Horsager et al. 2022).

In summary, marked frontal, temporal, and parietal cortical cholinergic deficits are correlated with falls and freezing of gait in patients with PD. Although M1 undergoes some degree of cholinergic denervation in PD, the assessment of the severity of changes in other motor cortical regions and their impact upon parkinsonian motor features necessitate further studies.

### Neurotransmitters

#### Glutamate

Most of the glutamatergic innervation of motor cortices arises from either the thalamus or other cortical regions. The thalamocortical system originates mainly from the basal ganglia- and cerebellar-recipient nuclei of the ventral motor thalamus, with lesser contributions from the rostral and caudal intralaminar nuclei (McFarland and Haber 2002; Guo et al. 2018; Shepherd and Yamawaki 2021; Villalba et al. 2021; Chen et al. 2023). In rodents and primates, the thalamic inputs profusely arborize in deep and superficial cortical layers of M1, pre-SMA, SMA, PMC, and CCz (McFarland and Haber 2002; Guo et al. 2018; Shepherd and Yamawaki 2021; Villalba et al. 2021; Chen et al. 2023).

Recent studies showed a significant breakdown of the thalamic innervation of cortical layer V in M1 of 6-hydroxydopamine- (6-OHDA-) treated parkinsonian mice and in MPTP-treated NHPs (Villalba et al. 2021; Chen et al. 2023). In both species, the density of terminals labeled with antibodies targeting the vesicular glutamate transporter 2 (vGluT2), a specific marker of glutamatergic terminals of thalamic origin, was significantly decreased in deep layer V (Fig. 2E–H). In mice, the thalamic denervation was accompanied by decreased density of dendritic spines on basal dendrites of pyramidal tract (PT) neurons, but not intratelencephalic (IT) corticofugal neurons (Chen et al. 2023).

It is possible that altered basal ganglia output to the thalamus and excessive activation of N-methyl-D-aspartate (NMDA) receptors at thalamocortical synapses on PT neurons contributes to the decreased thalamocortical innervation (Chen et al. 2023) (see further details below). Especially in parkinsonian monkeys, the loss of thalamocortical innervation may also result from the (known) degeneration of caudal intralaminar thalamic nuclei (Henderson et al. 2000; Henderson et al. 2001; Halliday 2009; Smith et al. 2014; Villalba et al. 2014; Villalba et al. 2019), which preferentially innervate the deep layers of M1 (Parent and Parent 2005).

Many unanswered questions remain before these observations can be translated to the human disease. Is there a breakdown of the thalamocortical projection to deep cortical layers of motor cortices in PD patients? If so, when does it occur during the development of parkinsonism? Does it affect specific subsets of PT corticofugal neurons? Is it related to a loss of thalamocortical terminals or a downregulation of their content in vGluT2? What cellular and synaptic mechanisms contribute to the development and maintenance of this neuroplastic event? Can it be reversed with antiparkinsonian therapies? Answers to these questions will help us to better understand if and how disrupted thalamocortical communication contributes to the development of parkinsonian motor signs.

#### GABA

The main source of GABA to motor cortices is the rich network of inhibitory cortical interneurons. Functional dysregulation of this network is well documented in patients with PD, with evidence for reduced GABA-A receptor-mediated short-interval intracortical inhibition (SICI, see below) in M1, correlating with disease severity (Kacar et al. 2013; Rothwell and Edwards 2013). There is limited information available on changes of GABA levels in the different motor cortical regions of PD patients. In their magnetic resonance spectroscopy study, van Nuland and colleagues found that reduced M1 GABA may contribute to the severity of parkinsonian motor signs (van Nuland et al. 2020). The same study showed that neither the phenotype of PD (tremor-dominant vs akinetic-rigid) nor the use of dopaminergic medications affected M1 GABA levels, raising doubts about the relevance of dysfunctional intracortical inhibitory GABAergic networks to PD pathophysiology. To further address this issue, Chu and colleagues (Cherian et al. 2024) recently assessed changes in the network of parvalbumin (PV)-containing GABAergic interneurons in M1 of 6-OHDA-treated mice and found that the loss of midbrain dopaminergic neurons does not affect the number, morphology, and physiology of these neurons in layer V in parkinsonian mice (Cherian et al. 2024).

To our knowledge, there is no other detailed quantitative stereological analysis of changes in the number of GABAergic interneurons in the motor cortices of PD patients or animal models of the disease, but in situ hybridization studies showed decreased neuronal expression of PV and glutamic acid decarboxylase in the prefrontal cortex of PD patients (Lanoue et al. 2010; Lanoue et al. 2013). Given the rodent data discussed above (Cherian et al. 2024), PV-positive interneurons may be differentially affected in specific cortical regions. Future neuropathological and functional studies of different populations of GABAergic interneurons are needed to better understand the role played by intracortical GABAergic networks dysfunction in PD.

### Pathology of motor cortices in PD

#### Morphometric, neuronal, and dendritic spine pathology

Neither traditional anatomical studies of postmortem material from patients with advanced PD (Halliday et al. 2005) nor conventional structural MRI studies have provided strong evidence favoring a reduction in motor cortices volume (Pedersen et al. 2005; Pyatigorskaya et al. 2014; Sterling et al. 2017; Fu et al. 2022). However, surface-based morphometry analyses of MRI scans have suggested cortical thinning of M1 as well as reduced gyrification of pre- and postcentral gyri in patients with advanced PD (Hutton et al. 2008; Hutton et al. 2009). Within-patient longitudinal studies have demonstrated an association between the progression of this gyrification loss and disease severity (Koshimori et al. 2015; Sterling et al. 2016; Li et al. 2020; Mitchell et al. 2021). In addition, diffusion tensor imaging studies revealed reduced fractional anisotropy in the subcortical white matter of M1 and the corpus callosum in patients with rapid eye movement (REM) sleep behavior disorder who converted to early PD after 2-year follow-up, suggesting deterioration of the integrity of white matter tracts during this period (Fu et al. 2022). M1 white matter damage associated with decreased concentrations of myelin proteins has been found in patients with advanced PD (Fu et al. 2022).

Microscopic morphometric analyses of pyramidal neurons in rodent and primate models of PD have reported dendritic spine loss in the prefrontal cortex (Solis et al. 2007; Elsworth et al. 2013). In primates, these changes were found in partially DA-denervated, motor-asymptomatic animals that displayed impairments in executive functions. The findings in the motor cortices are again more subtle. In fact, researchers found no change in the density, but an increase of the volume of individual dendritic spines on IT and PT neurons in M1 of 6-OHDA-treated rats, which was further accentuated in animals with L-DOPA-induced dyskinesias (Nishijima et al. 2018).

**Fig. 3:**
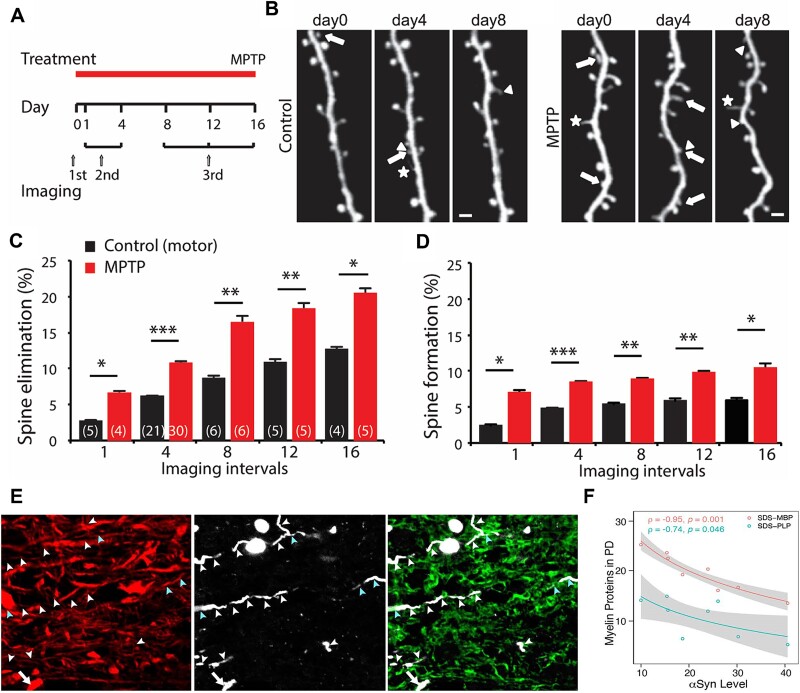
Dendritic spine pathology and myelination changes in parkinsonian mice. A–D) Spine dynamics in M1 in MPTP-treated mice. Neurons were identified by expression of yellow fluorescent protein (Thy1-YFP-H line) using trans-cranial two-photon laser scanning microscopy to study the growth or pruning of dendritic spines over time in controls and parkinsonian mice. A) illustrates the timeline of treatment and imaging. B) depicts imaged spines at 0, 4, and 8 days in control and MPTP-treated mice. Arrows: spines eliminated; arrowheads: spines formed; asterisks: filopodia. Scale bar: 2 mm. C, D) Bar graphs showing the spines eliminated (C) or formed (D) at different time points (as shown in A). See Guo et al. (2015) for more details. E, F) Myelin and axonal pathologies in white matter underlying the primary motor cortex in advanced PD cases. E) shows immunofluorescence labeling of axons (NFL, left panel), alpha-synuclein-containing neurites (P-αsyn, middle panel), and myelin basic protein (MBP, right panel). The white arrowheads indicate segments of axons in which P-αsyn displaced the longitudinal NFL and MBP labeling, suggesting myelination damage of some axonal profiles. The blue arrowheads point at P-αsyn+/NFL+/MBP+ axonal segments. F) shows negative Spearman rank correlations between the levels of myelin proteins (MBP and myelin proteolipid protein-PLP) and α-synuclein in PD cases. Gray zones indicate 95% confidence intervals that are automatically calculated using the predicted values for the line of best fit (see (Fu et al. 2022) for more details). Figure 2A–D reproduced from Guo et al. (2015), and E) and F) appeared in Fu et al. (2022). Both components are used with permission.

In addition to the frank loss of dendritic spines in M1, their turnover may also be increased in the (acute or subacute) parkinsonian state, as was shown in mice (Fig. 3) (Guo et al. 2015). Similar changes can also be induced with focal M1 DA depletion, indicating that the mesocortical DA system may locally regulate dendritic spine plasticity in M1. In the same study, the authors showed that these changes disrupt long-term potentiation (LTP), perhaps related to deficits in motor learning in these animals (Molina-Luna et al. 2009; Hosp et al*.* 2011b; Hosp and Luft 2013; Guo et al. 2015; Leemburg et al. 2018; Cousineau et al. 2022). D1 receptor blockade in M1 appears to promote spine loss, whereas D2 receptor blockade increases spine formation (Guo et al. 2015).

Thus, PD may be associated with early changes in the organization of white matter tracts underlying M1 and with cortical thinning of this region. Combined with the increased turnover of dendritic spines, this may disrupt synaptic integration and information transfer in M1, thereby contributing to the pathophysiology of motor signs and M1-mediated motor learning deficits associated with PD (Hosp et al*.* 2011b; Hosp and Luft 2013; Guo et al. 2015; Fu et al. 2022).

#### Lewy pathology/alpha-synuclein accumulation

In most forms of sporadic and genetic PD (except patients with Parkin mutation [Kitada et al. 2023]), Lewy pathology involving the motor cortex appears relatively late in PD, occurring only in stage 4 of the 6-stage Braak pathology staging system (Hurtig et al. 2000; Braak et al. 2003; Braak et al. 2004; Braak et al. 2006b; Dickson et al. 2010; Goedert et al. 2012; Halliday et al. 2012; Boeve 2013; Del Tredici and Braak 2016; Uchihara and Giasson 2016). Lewy pathology affects predominately pyramidal neurons in layers V and VI (Wakabayashi et al. 1995; Hurtig et al. 2000; Fu et al. 2022). The entorhinal cortex, anteromedial temporal cortex, and hippocampus are more severely affected (Dickson et al. 1994; Braak, de Vos, et al. 2006a; Braak and Del Tredici 2009) than high-order sensory association areas and prefrontal cortex, first-order sensory association areas, premotor fields, and primary sensory and motor areas (Braak et al. 2003; Braak and Del Tredici 2009).

In a recent study, Fu et al. (2022) reported more subtle Lewy pathology in M1, affecting the subcortical white matter, leading not only to damage of myelin sheaths around axons but also an enlargement of myelinating oligodendrocytes, and an increased density of their precursors (Fig. 3E). They also reported that the concentration of phosphorylated alpha-synuclein correlates with reduced subcortical white matter myelin proteins in M1 of PD patients (Fig. 3F).

Given the details of timing, location, and severity of the cortical involvement, Lewy pathology in cortical neurons may be most closely related to impairments of cognition, but may not be the primary driver of motor disturbances early in the disease. However, as discussed above, synuclein aggregation in the subcortical white matter may disrupt processing in M1, at least in advanced stages of the disease (Fu et al. 2022). Alpha-synuclein PET imaging (Xiang et al. 2023) may help us to study further the impact of regional Lewy pathology on behavior in the near future.

## Electrophysiological changes related to motor abnormalities in PD

Several technical approaches have been used to study parkinsonism associated changes in electrophysiologic activity of cortical areas, including electroencephalography (EEG), electrocorticography (ECoG), magnetoencephalography (MEG), and recordings of single and multiple cells in vivo and ex vivo. In addition, cortical networks in parkinsonian patients have been interrogated with transcranial magnetic stimulation (TMS). Some of the salient results of studies using these approaches will be presented below, focusing on changes that appear to be linked to the motor signs of the disease.

### E‌EG biomarkers for PD

Normal movement is associated with typical sequences of beta-band (13 to 30 Hz) desynchronization and synchronization of frontal EEG (Pfurtscheller et al. 1998; Kühn et al. 2006; Feingold et al. 2015; Khanna and Carmena 2017; O'Keeffe et al. 2020; Bonaiuto et al. 2021). PD patients suffer from general background EEG slowing (reviewed by Silberstein et al. 2005), and excessive overall beta band oscillatory synchronization (Bočková and Rektor 2019). EEG studies also showed greater cortico-cortical coherence in the beta-band, correlating with the severity of motor signs of PD (Silberstein et al. 2005), and linked to the loss of DA in the striatum (Waninger et al. 2020).

The excessive beta band synchrony and especially the loss of temporal modulation of beta band activity may have pathophysiologic relevance (Defebvre et al. 1998; Brown and Marsden 1999; Wang et al. 1999; Magnani et al. 2002; Devos et al. 2003; Chung et al. 2018). These changes correlate with clinical motor scale ratings (Karimi et al. 2021). In contrast, suppression of the excessive synchronization by the performance of simple cueing tasks (Tosserams et al. 2022), or by treatment with levodopa (Bočková and Rektor 2019) or deep brain stimulation (DBS) (Conti et al. 2022), is associated with improvements in motor function.

### Studies of cortical ECoG and local field potentials in PD

One of the benefits of the study of ECoG signals over scalp EEG signals is that the spatial precision is greater and that the signals contain significant power at higher frequencies, allowing for detailed studies of gamma-band activities (in addition to lower frequencies).

**Fig. 4:**
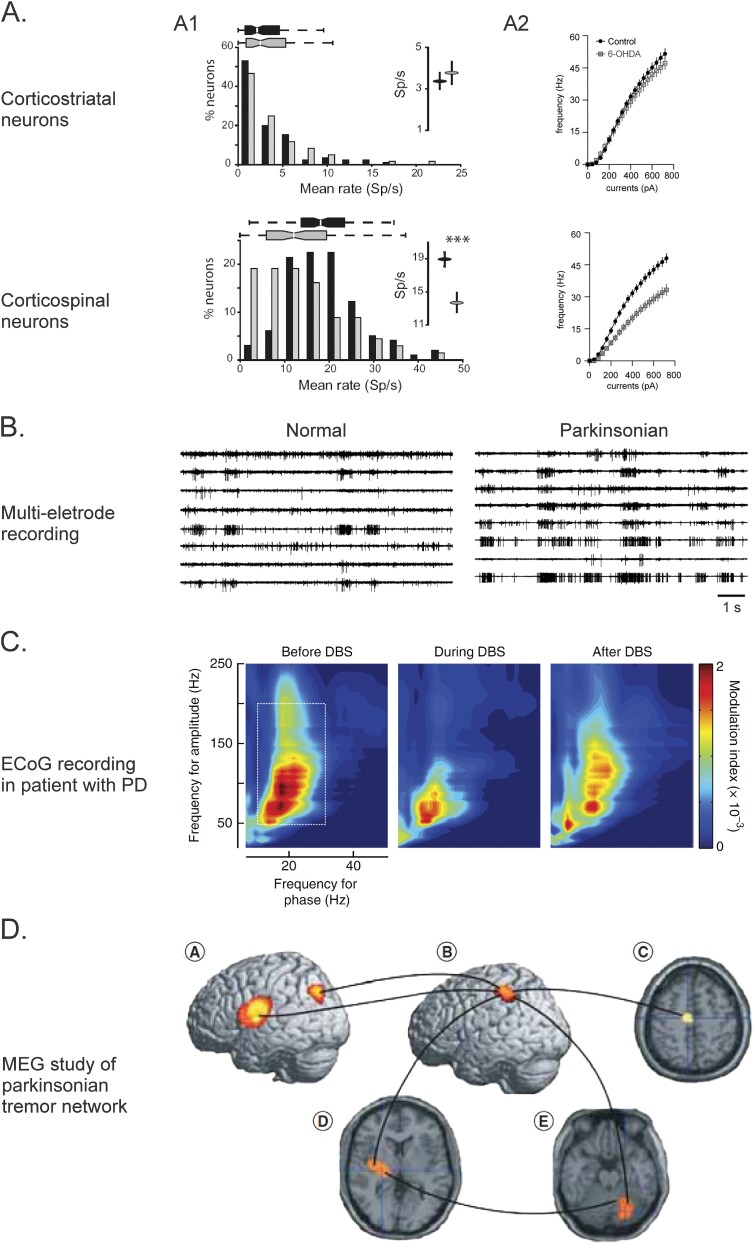
Examples of recordings of electrophysiologic and magnetoencephalographic recordings of abnormal M1 activity in the parkinsonian state. A) In vivo single-cell recordings in M1 of parkinsonian NHPs (A1) and in brain slices of parkinsonian mice (A2) demonstrate that corticostriatal neurons show no changes in firing rates (primates) (A1, top) or the spiking response to current injections (their excitability) (A2, top), while PT neurons fire at lower rates (A1, bottom), and are less excitable (A2, bottom). B) In vivo multielectrode recordings in M1 in normal and parkinsonian NHPs demonstrate that cortical neurons show a greater tendency to discharge in synchronized bursts. C) The images show an analysis of recordings of ECoGs in parkinsonian patients, made while the patients underwent DBS lead placement procedures. Shown is the (color-coded) modulation index, a measure of the coupling between the phase of low-frequency oscillations and the amplitude of high-frequency oscillations. Parkinsonian patients show strong phase-amplitude coupling between beta- and gamma oscillatory ECoG activity that is ameliorated by DBS. D) The figure shows an oscillatory network associated with parkinsonian resting tremor, as identified with MEG and dynamic imaging of coherent sources analysis. As demonstrated, M1 (circle B) is coupled to other cortical areas, including the lateral premotor cortex (circle A) and the SMA (circle C), with diencephalic areas (circle D), and with the cerebellum (circle E). Part A1 of the figure reproduced from Pasquereau and Turner (2011), part A2 reproduced from Chen et al. (2023), part B comes from Goldberg et al. (2002), and part C from Hemptinne et al. (2015) and part D from Timmermann et al. (2007). All figures are used with permission. Part B is covered by copyright 2002, Society for Neuroscience.

Starr et al. found that broad-band gamma-band activity and movement-associated changes in beta-band activity were exaggerated in M1 of parkinsonian patients, compared to patients with dystonia or essential tremor (Crowell et al. 2012). In later studies (de Hemptinne et al. 2013; de Hemptinne et al. 2015), PD was found to be associated with an increased coupling of beta- and gamma-band activities in ECoG signals recorded in M1. This finding suggests that spiking activity in M1 (reflected by broadband gamma-band activity) may be excessively synchronized to the phase of beta-band network oscillations (i.e. phase-amplitude coupling, PAC). The enhanced synchronization can be reversed by antiparkinsonian interventions, including levodopa treatment and DBS (Fig. 4C) (Swann et al. 2015; Miller et al. 2019; Eguchi et al. 2021), or by movement execution (Cross et al. 2021).

While beta-band activity may be “antikinetic,” it has been argued that gamma-band oscillations are “prokinetic,” reflecting local neural activity in sensorimotor areas. Interventions that reduce cortical beta-band synchrony (such as DBS of the subthalamic nucleus [STN]), and the entrainment of cortical gamma-band activity to beta band activity, increase gamma-band power in motor and premotor cortices (Muthuraman et al. 2020).

ECoG signals have also been used to compare some of the pathophysiologic signatures of different motor symptoms of PD. One area of interest has been the study of tremor. In general, the cortical electrophysiological abnormalities in tremor, including reduced beta band power, cortico-cortical coherence, and PAC (Qasim et al. 2016), are substantially different from those associated with bradykinesia (Lauro et al. 2023). A popular hypothesis describing the emergence of tremor in PD poses that epochs of tremor are triggered by abnormal basal ganglia output to the thalamus and are then maintained by oscillatory activity (Lauro et al. 2021) involving M1 and the ventral intermedius nucleus of the thalamus (Madelein van der Stouwe et al. 2020), an element of the cerebellar outflow pathways.

Another area of interest is the cortical involvement in the pathophysiology of parkinsonian freezing of gait. ECoG studies showed greater theta-frequency coupling of M1 ECoG signals to local field potentials (LFPs) recorded from the STN (Louie et al. 2022), and greater beta/gamma PAC in M1 (Yin et al. 2022) during episodes of freezing, suggesting a role of the STN and of momentary worsening of cortical PAC during freezing episodes.

LFP and ECoG recordings in MPTP-treated NHPs showed that synchronized oscillations in subcortical and cortical recordings emerge as the animals develop parkinsonism. Devergnas et al. (2014) found that the severity of parkinsonism correlated with increases in spectral power and coherence between M1 ECoG and internal pallidal LFP signals in the alpha- and low beta-bands, with M1 changes occurring early during the development of parkinsonism. A more recent study showed that the incidence, duration, and amplitude of brief cortical increases in beta band power (“beta-bursts”) did not change in the parkinsonian state, but that beta bursts in M1 tended to co-occur with beta-bursts in subcortical structures (Yu et al. 2021). Of note, evidence for substantial interindividual variability between animals with similar levels of parkinsonism suggest that the observed changes in cortical beta bursts may not be central to the pathophysiology of parkinsonism. This hypothesis is supported by the dissociation between an early expression of bradykinesia-like motor deficits and the delayed development of beta band activity in M1 in parkinsonian rodents (see below) (Brazhnik et al. 2021).

### MEG studies in parkinsonian patients

MEG studies can inform us about parkinsonism-associated cortical oscillatory behavior as well as cortico-cortical and cortico-subcortical connectivity, providing better spatial resolution than EEG studies (and brain-wide coverage, as opposed to ECoG studies). MEG studies have shown that resting cortico-cortical connectivity across theta, alpha, and beta bands is increased in patients with PD (Stoffers et al. 2008; Vardy et al. 2011; Heinrichs-Graham et al. 2014a; Heinrichs-Graham et al. 2014b; Cao et al. 2015; Prokic et al. 2019). Studies in patients at early stages of the disease emphasized especially an increased overall amount of beta band oscillations and an inability to functionally reduce it (Pollok et al. 2012; Hall et al. 2014), with some of these changes considered as being compensatory (Pollok et al. 2013; Disbrow et al. 2022; Wiesman et al. 2023). MEG studies confirmed that the typical desynchronization–resynchronization sequence of movement-related cortical activities (see above) is disrupted in the parkinsonian state (Meissner et al. 2018; Vinding et al. 2019), along with a demonstration of a generally lower beta-*burst* rate in PD patients (Heideman et al. 2020; Vinding et al. 2020; Vinding et al. 2024). Recent studies have also demonstrated that there are changes in cortical gamma/beta band phase amplitude coupling (Tanaka et al. 2022; Mertiens et al. 2023).

Combining MEG techniques with electromyogram (EMG) recordings has allowed study of the cortico-muscular coherence (CMC). These studies showed that the beta-band CMC involving M1 negatively correlates with akinesia and rigidity (Hirschmann et al. 2013; Zokaei et al. 2021). Similarly, the coherence between M1 and STN activity can be studied using a combination of MEG with LFP recordings in the STN obtained from implanted DBS electrodes. In these studies in parkinsonian patients, coherent activity was found to occur in the beta range between the STN and the ipsilateral sensorimotor and premotor cortex, and in the alpha range between the STN and the ipsilateral temporal lobe (Hirschmann et al. 2011; Litvak et al. 2011; van Wijk et al. 2016), although this was not confirmed by others (Bock et al. 2013). A more detailed analysis showed that the coherence between the STN and the SMA in the high beta band correlates with the (functional) density of the corticosubthalamic projection and may thus reflect a direct influence of SMA on STN activities (Oswal et al. 2021).

Dopamine replacement therapy (further) increases cortical connectivity in parkinsonian patients (Stoffers et al. 2008), with some MEG studies documenting treatment-induced increases in the cortical alpha band (Ozkurt et al. 2020) or the high-beta band (Cao et al. 2020). Increased coherence with the medial and orbitofrontal cortex, perhaps related to the cognitive side effects of levodopa treatment (Sharma et al. 2021), has also been reported.

Most MEG studies have suggested that STN-DBS augments gamma- and suppresses alpha/beta cortical oscillations (Cao et al. 2015; Cao et al. 2017; Abbasi et al. 2018; Luoma et al. 2018; Boon et al. 2020; Boon et al. 2023). Other studies suggested DBS-related hypersynchrony in high beta- and gamma bands in frontal and prefrontal, occipitoparietal, and medial temporal cortices (Wang et al. 2022) and STN-DBS-induced normalizations of cortical beta-burst dynamics in the sensorimotor cortex (Pauls et al. 2022). The relationship between these findings and the clinical effects of STN-DBS is not clear (Luoma et al. 2018; Boon et al. 2020).

### Insights of motor cortical dysfunction in PD from TMS studies

TMS is a noninvasive technology for functional modulation of brain activity. It generates an electromagnetic field that penetrates through the skull, induces an electric field in brain tissues, and modulates neuronal firing.

By positioning TMS coils over M1, *single*-pulse TMS can be used to assess the corticospinal excitability, as reflected by the size and kinetics of motor-evoked potentials (MEPs) in EMG recordings from muscles reached by the activated corticofugal fibers. With few exceptions, the available studies have reported normal MEP thresholds in PD patients (Lefaucheur 2005; Bologna et al. 2016; Vucic et al. 2023). However, the timing of motor cortical activation and deactivation following TMS stimulation in early-disease patients is slowed (Chen et al. 2001). Such changes may reflect corticospinal excitability changes in early-stage PD, consistent with changes demonstrated by other modalities (see other sections).

*Repetitive* TMS (rTMS) protocols have also been applied to study cortical plasticity in PD (Bologna et al. 2016; Vucic et al. 2023). In healthy individuals, high frequency rTMS of M1 (e.g. at 5 Hz) induces a progressive increase of the size of MEPs following each stimulus within the stimulation train (Berardelli et al. 1998). This facilitatory effect is much smaller in untreated patients with PD (Gilio et al. 2002), even in early-stage PD (Kishore et al. 2012), and not universally responsive to DA replacement therapy (MacKinnon et al. 2005; Chu et al. 2009). It has been hypothesized that the decreased cortical inhibition in SICI studies in PD patients might be due to impaired cellular excitability and/or inhibitory synaptic transmission of cortical GABAergic interneurons (Ridding et al. 1995; MacKinnon et al. 2005; Ni et al. 2013; Vucic et al. 2023).

### Single-cell recordings in animal models of parkinsonism

Early single neuron recording studies in the M1 in MPTP-treated parkinsonian NHPs (Doudet et al. 1990) did not reveal significant changes in spontaneous neuronal activity, but demonstrated that the timing of movement-related activity was changed, with responses starting earlier, ramping up more slowly, and lasting longer than in the normal state (but see Mandir and Watts 1990). Other studies found that MPTP administration led to an alteration in the timing of premovement activity in the SMA (Watts and Mandir 1990). In later studies, M1 neurons were found to (spontaneously) discharge in long synchronized bursts (Fig. 4B) (Goldberg et al. 2002). Most authors documented that M1 and SMA neurons respond with less specificity to passive limb movements (Doudet et al. 1990; Watts and Mandir 1990; Goldberg et al. 2002).

In meticulous recording studies by Pasquereau and Turner (Pasquereau and Turner 2011, 2013; Pasquereau et al. 2016), using healthy and MPTP-treated monkeys, the authors found that the spontaneous activity of PT neurons (identified by antidromic stimulation of the pons) was reduced, with an increase in bursting, and greater irregularity in firing, while the firing of corticostriatal neurons was not altered (Fig. 4A1). Similar to the findings of earlier studies mentioned above, they also demonstrated changes of the timing of responses to muscle stretch and a reduction of directional selectivity of such responses (Pasquereau and Turner 2013). In studies of neuronal activation in relation to active movements, the timing of M1 PT neuron activation was disturbed (reminiscent of the changes previously described in SMA [Watts and Mandir 1990]) and the responses to active movements were reduced (Pasquereau et al. 2016).

In studies of motor cortical activity in rodents, the firing frequency of putative layer 5 pyramidal neurons in M1 was found to be reduced in animals with acute haloperidol-induced parkinsonism (Parr-Brownlie and Hyland 2005) or chronic parkinsonism induced by 6-OHDA treatment (Parr-Brownlie et al. 2022; Sun et al. 2023). By simultaneously recording LFPs and single-cell activity in hemiparkinsonian rats, Brazhnik and Walters showed that abnormally synchronized beta-band oscillations between M1 and the motor output nucleus of the rodent basal ganglia, the substantia nigra pars reticulata (SNr), emerged gradually following DA depletion (Brazhnik et al. 2012), likely linked via thalamocortical connections (Brazhnik et al. 2016). These findings are consistent with the findings of excessive coupling between M1 and subcortical regions in parkinsonian NHPs and persons with PD (Gatev and Wichmann 2009; Shimamoto et al. 2013).

Following up on the studies by Pasquereau et al. (see above), an ex vivo (patch-clamp) study in mice rendered parkinsonian by unilateral treatment with 6-OHDA focused again on differences between corticofugal systems in M1 (Chen et al. 2021). In this study, PT neurons were compared with IT neurons, which provide not only contralateral projections to the corresponding cerebral cortex but also a large portion of the corticostriatal inputs in rodents. Chen et al. (2021) found that the intrinsic excitability of PT neurons, but not that of IT neurons, was reduced in the parkinsonian state (compared to the normal state, Fig. 4A2). They documented that these cells showed a more depolarized threshold and greater width of action potentials and that they did not sustain high-frequency firing. Mechanistically, several changes were identified, including impaired functions of persistent Na^+^-channels and of large-conductance, Ca^2+^-activated K^+^-channels. Another key finding of this study was that the observed changes were not rescued by acute DA receptor activation, suggesting that the electrophysiologic shift may not (only) be caused by cortical DA loss, but may result from other subacute or chronic adaptations affecting the basal ganglia-thalamocortical circuitry. To explore such possibilities, the same group tested the effects of chemogenetically suppressing basal ganglia output on the cellular excitability of cortical PT neurons in mice with 6-OHDA lesions (Chen et al. 2023) and found that the chemogenetic circuit manipulation did not rescue the impaired excitability of PT neurons of DA-depleted mice. Additional studies are needed to further address the impact of the observed changes of intrinsic excitability of M1 PT neurons on M1 neuronal circuits (see below).

### Overall view of electrophysiologic abnormalities of motor cortices in PD

The experiments mentioned above have shown that cortical activity changes in the parkinsonian state affect highly specific neuron types and that such changes may, at least in part, result from plasticity of thalamocortical connections. The existing results also suggest that parkinsonian motor signs (akinesia/bradykinesia and tremor) involve multiple (cortical and subcortical) circuit dysfunctions instead of single network mechanisms. Another important insight is that dynamically changing synchronized oscillatory phenomena, including changes in the temporal structure of beta-band oscillations, or their entrainment of gamma oscillations, are relevant for cortical dysfunction in PD. It is reasonable to speculate that alterations of these interactions could disturb the highly orchestrated sequence of brain activation patterns that causes and accompanies behavior.

## In vivo imaging of motor function in PD patients

### Motor cortices at rest and with tasks

There has been a long-standing interest in understanding the impact of subcortical DA deficiency on the activity of motor cortices in PD patients. Combining imaging results with knowledge about the patient’s motor function, treatment history, and specific motor task demands, an invaluable window into the pathophysiology of PD begins to emerge.

**Fig. 5:**
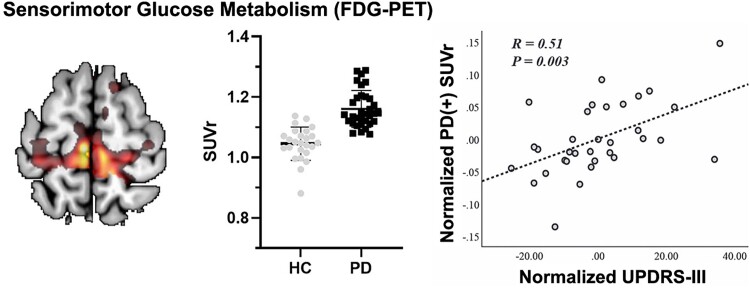
Examples of imaging studies in patients with parkinsonism. Left and middle panels: Increased FDG uptake in sensorimotor cortex of PD patients compared to healthy controls. Right panel: A significant positive correlation between sensorimotor cortical FDG uptake and UPDRS III score in PD patients. From Zang et al. (2022) and used with permission.

One of the most consistent imaging observations of PD patients “at rest” and off medication has been that M1 glucose metabolic activity or cerebral blood flow (CBF) as measured with the PET ligands ^18^F-deoxyglucose (FDG) or H_2_^15^O, respectively, are elevated (Feigin et al. 2001; Hershey et al. 2003). The magnitude of the apparent increase in M1 metabolism has been correlated with the severity of tremor and motor scores on the Unified Parkinson’s Disease Rating Scale (UPDRS; Fig. 5). Further, longitudinal studies have shown a correlation between worsening motor scores and increasing metabolism (Huang et al. 2007; Mure et al. 2011; Prodoehl et al. 2013; Matthews et al. 2018; Zang et al. 2022). When combined with measures from the basal ganglia, the FDG metabolic signature in M1 is sufficiently robust to discriminate PD from other parkinsonian syndromes or healthy controls (Matthews et al. 2018; Gu et al. 2019). M1 metabolic increases can be reduced toward normative values with either L-DOPA treatment or thalamic/STN-DBS (Feigin et al. 2001; Mure et al. 2011). Similarly, the elevated resting CBF in M1 of PD patients (see above) is reduced with L-DOPA or STN-DBS treatment (Hershey et al. 2003; Haslinger et al. 2005).

A more complex picture emerges when the functional activity within motor cortices (M1 and premotor regions) is interrogated while PD patients perform motor tasks during PET imaging, CBF measurements, or fMRI Blood Oxygen Level Dependent (BOLD) response imaging. Here, we focus on the recruitment of M1 and SMA. The motor tasks that have been used typically require paced finger tapping, dynamic control of grip force, or joystick movements that are either cued or self-generated with single or sequential targets. The studies mentioned below achieved similar motor performance between patients and healthy controls. Whether activation was measured by PET or fMRI, the most common finding across early studies was underactivation of the SMA compared to healthy control subjects (Jenkins et al. 1992; Playford et al. 1992; Sabatini et al. 2000; Haslinger et al. 2001; Spraker et al. 2010; Burciu et al. 2015). Underactivation of M1 in these types of tasks was also described (Turner et al. 2003; Spraker et al. 2010; Burciu et al. 2015; Neely et al. 2015; Planetta et al. 2015), although in a few studies, the task performance in PD patients was associated with elevated activation of M1 and a concomitant underactivation of SMA (Sabatini et al. 2000; Haslinger et al*.* 2001; Yu et al. 2007) or even a combined increase of activation in both SMA and M1 (Thobois et al. 2002). These outliers may reflect a compensatory response in PD patients to sustain motor performance in particular tasks and differences in task demands or could be a result of differences in medication history across the tested patient groups. Of note, both under- and overactivation of SMA and/or M1 have been modified toward a more normal pattern of activation after treatment with L-DOPA, pallidotomy, or STN-DBS (Jenkins et al. 1992; Limousin et al. 1997; Haslinger et al. 2001; Thobois et al. 2002; Grafton et al. 2006).

How might the discordance between the commonly observed PD-related *increase* of M1 metabolism at rest and the underactivation in M1 and SMA with tasks be reconciled? One important fact to consider is that most PD patients have significant overt or covert motor features at rest that may drive M1 metabolism. Several motor features, such as tremor (Neely et al. 2015), freezing of gait, rigidity, or akinesia (Sabatini et al. 2000), correlate with *resting* functional activity in SMA and/or M1 (Yu et al. 2007). In other words, while the patient may be “at rest,” the motor cortices are active and likely contribute to (ongoing) motor symptoms, such as akinesia and tremor. This is consistent with the presence of pathologic oscillations at rest (described above) in motor cortices with a resultant elevated resting metabolism or CBF. As such, this elevated resting activity would be associated with a limited (relative) range within which the cortex would “activate” with tasks. Alternatively, imaging experiments with motor tasks showing underactivation in SMA and or M1 in parkinsonian patients have been interpreted as being a consequence of either excessive pallido-thalamic inhibition and a corresponding lack of cortical recruitment and/or a consequence of pathologic thalamocortical oscillations that would also interfere with motor cortical recruitment irrespective of baseline activity levels (Grafton and DeLong 1997; Grafton 2004).

### The basal ganglia-thalamocortical motor network

Imaging can be used in PD patients to assess changes of functional connectivity between cortical and subcortical structures and activity in motor cortices. Focusing on areas with direct anatomic connections with M1, there is evidence for reduced functional connectivity between M1 and the thalamus in PD patients who were scanned in the off medication state (Shah et al. 2016). This alteration can be modified by pallidotomy or physical therapy (Grafton et al. 2004; Shah et al. 2016). These findings are consistent with the classic model of PD whereby increased basal ganglia inhibition onto the thalamus leads to reduced thalamocortical facilitation.

The functional connectivity between M1 and the putamen, as measured by rsfMRI, is also reduced, even in early-stage PD (Helmich et al. 2010; Luo et al. 2014). As this connectivity decreases over time, there is worsening of parkinsonian motor scores in longitudinal studies (Steidel et al. 2022). In contrast, there is evidence from rsfMRI for enhanced connectivity between M1 and STN that increases with disease severity, suggesting a potential involvement of the cortico-subthalamic pathway (Baudrexel et al. 2011; Kurani et al. 2015). This too is modulated toward a more normal pattern with treatment (Kahan et al. 2014).

### Extended multiscale network dynamics

Expanding on the network approach, both linear and nonlinear estimates of functional connectivity measured at slow frequencies by rsfMRI have demonstrated abnormalities in PD patients consistent with what has been described at higher frequencies by EEG and other methods (Zhang et al. 2019; Sarmiento et al. 2020). These imaging studies have demonstrated decreased functional connectivity within the sensorimotor areas and between sensorimotor cortices and other cortical areas (Wu et al. 2011; Caspers et al. 2021). Within the sensorimotor network, there is an overall reduction of connectivity, although causality models of specific connections showed an increased input from the dorsal premotor cortex to M1, inversely related to PD motor severity (Hao et al. 2020). Alterations of cortical functional connectivity, whether measured by EEG or MEG (see above) or rsfMRI, are partially normalized with dopaminergic treatment and DBS (Evangelisti et al. 2019; Caspers et al. 2021; Zhang et al. 2021; Chu et al. 2023).

## From correlation to causality: the utility of multiscale simulation

Anatomical, functional, and imaging changes observed at the cortical level in the parkinsonian state have typically been treated as separate elements. Integration of these multimodal changes is necessary for providing an understanding of how changes at the pharmacological and cellular levels lead to systems pathophysiology. The use of cohesive multiscale neuronal modeling (MSM, a variety of computer simulation) can integrate this information. MSM is constantly developing, and it is best performed hand in hand with the use of experimental data such that new discoveries can be incorporated quickly into models. Model predictions can be tested in experiments (Lytton et al. 2017; Peng et al. 2021; Geerts et al. 2023).

Computer simulation of circuit brain dynamics has largely utilized highly reduced brain-region or whole-brain models. Instead of modeling cells, large brain regions are represented by individual equations that are then linked together to model activity of the brain in the parkinsonian state. Such models focus on the interactions between basal ganglia, STN, thalamus, and cortex in a setting of reduced DA input from the substantia nigra and have been used to better understand and plan DBS (Humphries et al. 2018) and to provide insights into the source of movement abnormalities. Whole-brain modeling was also used to demonstrate patterns of areal connectivity from fMRI, at scales involving several cubic millimeters of cortex over with temporal resolution of seconds (Jung et al. 2022) (see also above in the section on imaging abnormalities).

A limitation of these large-scale models is that they do not reach to the cellular and synaptic levels where pathological and neuroplastic changes occur or to the molecular level where pharmacological targets are provided. Some recent publications have tried to address these limitations by coupling large-scale areal models with smaller-scale neuronal network models in MSMs (Kerr et al. 2013; Meier et al. 2022).

**Fig. 6:**
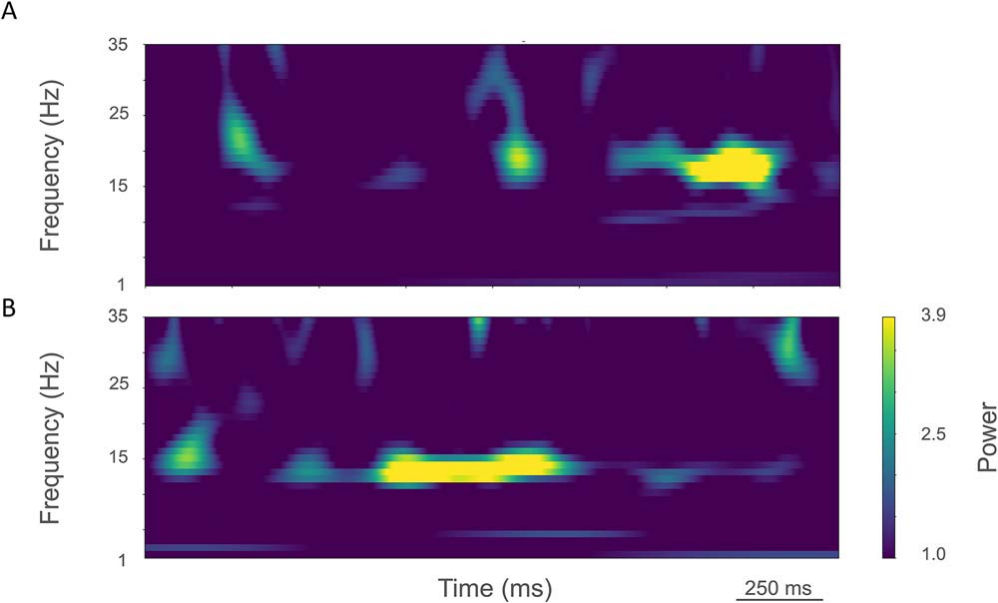
Spectrogram of LFPs in simulated M1. A) Control state. B) Parkinsonian state (modified from Doherty et al. 2024).

As noted above, electrophysiological studies have shown alterations in the intrinsic physiology of PT neurons in the M1 of 6-OHDA-treated mice (Chen et al. 2023). We have used detailed PT neuron simulations in the context of a full mouse M1 model to assess the demonstrated reduction in PT neuron excitability (Doherty et al. 2024). The *decreased* cell excitability actually produced *increased* cell activity in the network, along with increased beta band LFP oscillations which are a signature of the PD state (Fig. 6), and may also explain some of the seemingly paradoxical imaging abnormalities in the motor cortices (see discussion above).

## The motor cortex as a target of neuromodulation for PD treatment

### Insights from studies of subcortical DBS

**Fig. 7:**
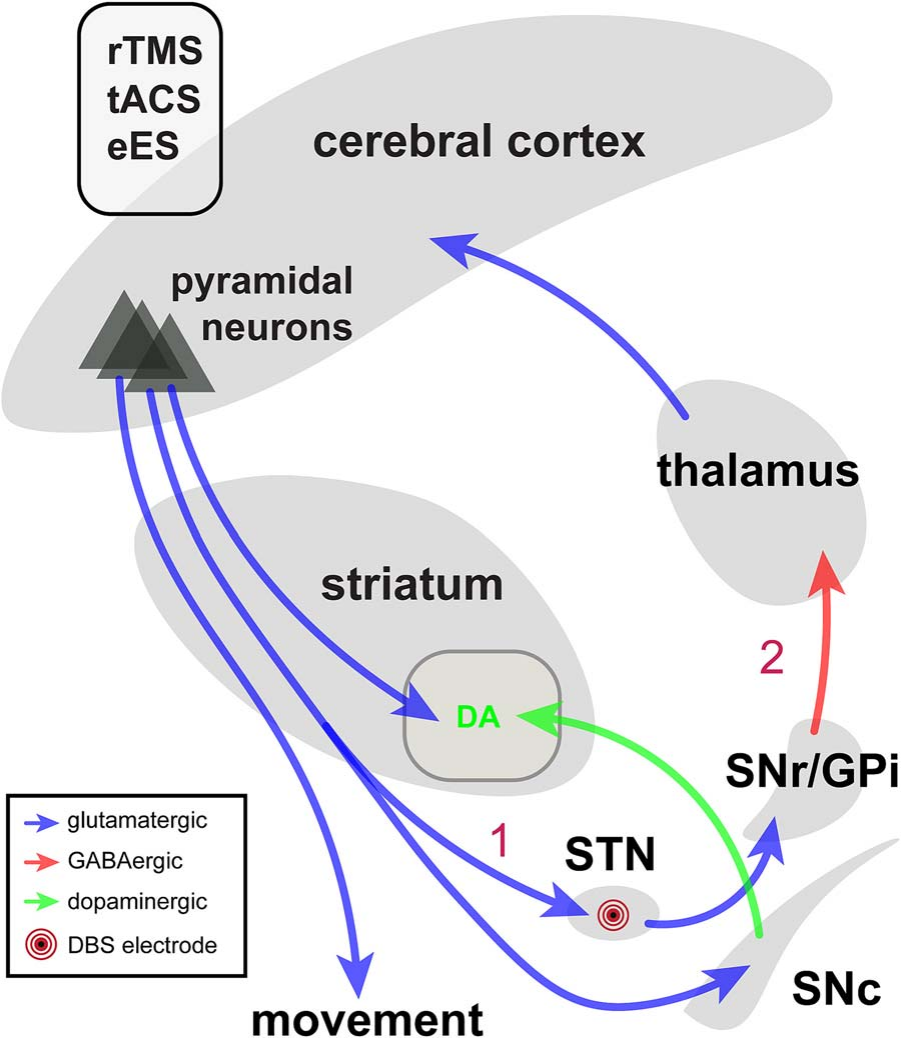
Simplified circuit diagram showing potential mechanisms of cortical neuromodulation for PD treatment. Cortical pyramidal neurons and local circuits can be functionally modulated by electric stimulation of basal ganglia structures (e.g. the STN) via 1) antidromic and 2) orthodromic circuits, by the less invasive direct rTMS, tACS modalities, or by epidural electric stimulation (eES) of the motor cortex. Concentric circles inside the STN indicate the DBS electrode.

Findings from electrophysiological recordings and functional imaging studies are compatible with the hypothesis that the therapeutic effects of STN or GPi DBS treatment may involve neuromodulation of cortical circuits. In studies involving human subjects, STN-DBS evokes short-latency potentials (occurring 2 to 3 ms after individual STN stimulation pulses) in M1, the premotor cortex, and the primary somatosensory cortex. These responses are likely mediated by antidromic activation of cortico-subthalamic axons (Fig. 7) (Kuriakose et al. 2010; Miocinovic et al. 2018; Irwin et al. 2020). Two recent studies have reported that the amplitude of DBS-evoked short-latency M1 potentials predicts the long-term clinical benefits of chronic DBS treatment in PD patients (Miocinovic et al. 2018; Irwin et al. 2020), indicating that antidromic activation of these pathways may contribute to (or at least correlate with) the therapeutic effects of DBS treatment.

Studies in parkinsonian NHPs (Devergnas and Wichmann 2011; Johnson et al. 2020) and rodents (Li et al. 2007; Dejean et al. 2009; Li et al. 2012; Sanders and Jaeger 2016) also support a role of motor cortex modulation in mediating the therapeutic benefits of STN-DBS. Taken together, clinical and preclinical studies suggest that cortical neuromodulation may contribute to the therapeutic effects of DBS and that some of the cortical effects of STN-DBS may be mediated via antidromic activation of the corticosubthalamic pathway.

As expected, STN/GPi DBS in NHPs and patients with PD was shown to also modulate cortical activity through *orthodromic* activation of basal ganglia–thalamocortical connections (Fig. 7) (Devergnas and Wichmann 2011; Miocinovic et al. 2018; Johnson et al. 2020). These effects (as opposed to antidromic effects) likely dominate the clinical effects of GPi-DBS, because the motor region of the GPi lacks strong direct cortical inputs (Johnson et al. 2009), although such inputs have, in fact, been described (Milardi et al. 2015; Smith and Wichmann 2015). Whether the differences in STN and GPi connectivity are an explanation for different L-DOPA requirements of patients who undergo these procedures remains completely undefined.

It is also worth noting that cortical responses to STN or GPi DBS are not homogenous in NHP studies (Johnson et al. 2009; Johnson et al. 2015; McCairn and Turner 2015), reflecting either the dynamic interaction between various circuits following the stimulation and/or the heterogeneity of pyramidal cortical neuron subtypes. It could also be that cortical interneurons contribute to the motor benefits produced by STN-DBS (Valverde et al. 2020).

### Electrical cortical stimulation

Relative to the currently favored DBS targets in the basal ganglia (STN, GPi), the motor cortex is more accessible for electrical stimulation, reachable by minimally invasive placement of epi- or subdural electrodes. Direct cortical neuromodulation could potentially be used to produce therapeutic effects by altering cortical excitability, disrupting excessive network synchronization and oscillation, or by suppressing pathologic PAC.

Early studies using epidural M1 stimulation in humans with PD reported minimal benefits (Gutierrez et al. 2009; Moro et al. 2011). A more recent long-term follow-up study reported, however, that extradural motor cortex stimulation was, in fact, safe and effective in managing overall parkinsonian complications and that it could lead to a gradual reduction in patient requirement for DA replacement therapy (Piano et al. 2021).

The therapeutic efficacy of epidural cortical stimulation also varied between NHP studies. An early study in MPTP-treated parkinsonian NHPs showed that high-frequency motor cortex stimulation reduced akinesia and bradykinesia, and normalized the neuronal activity in GPi and STN (Drouot et al. 2004), but another study, using more severely parkinsonian NHPs, found that such effects were only short-lasting and noticeable only when the animal engaged in specific tasks (Wu et al. 2007).

At least in early-stage PD, cortical stimulation may also increase DA signaling (Strafella et al. 2005). In support of this notion, a recent preclinical study found that optogenetic stimulation of glutamatergic pyramidal neurons in the M2 (roughly equivalent to the premotor cortex/SMA in NHPs; Fig. 1) of mice with partial striatal dopamine depletion increased the animals’ locomotor activity, mostly through an increase of DA levels consequent to the optogenetic activation of M2-SNc projection (Magno et al. 2019).

### Transcranial magnetic stimulation of the motor cortex

In addition to its use as a research tool (see above), the potential antiparkinsonian effects of M1-rTMS have also been assessed (Fregni et al. 2005; Elahi et al. 2009; Moro et al. 2011; Brys et al. 2016). High-frequency rTMS stimulation of M1 improved akinesia and bradykinesia in PD patients, but was less effective for the treatment of tremor or gait disturbances (Pascual-Leone et al. 1994; Mally and Stone 1999; Siebner et al. 1999; Shimamoto et al. 2001; Fregni et al. 2005; Brys et al. 2016). The durability of the antiparkinsonian rTMS effects appears to be highly variable, ranging from days to weeks (Zanjani et al. 2015). Moderate improvement of motor function in PD patients after rTMS of the SMA has also been reported (Hamada et al. 2008, but see Boylan et al. 2001).

As is the case for the therapeutic benefits of rTMS in general, the mechanism(s) that mediate these effects remain(s) unknown (Vucic et al. 2023). Conceivably, rTMS modulates cortical excitability via direct stimulation of PT corticofugal neurons, may regulate subcortical or cortical DA release, as discussed above (Shimamoto et al. 2001) (see also Kanno et al. 2004; Yang et al. 2010), or may alter cortical GABA levels and/or regulate synaptic plasticity (Ridding et al. 1995; MacKinnon et al. 2005; Ni et al. 2013).

### Transcranial alternating current stimulation of the motor cortex and other methods

Several studies have tested whether transcranial alternating current stimulation (tACS) of the M1 at (“pro-kinetic”) gamma frequencies could have antiparkinsonian effects in patients with PD. Guerra et al. (2020) demonstrated that gamma-band (70 Hz) tACS of M1, but not beta band (20 Hz) tACS, restored the impaired LTP-like plasticity in patients with mild to moderate PD (Guerra et al. 2020), independent of dopaminergic medication use, indicating that it may have acted by engaging nondopaminergic mechanisms (Guerra et al. 2023). Brittain et al. (2013) applied tACS over M1 in patients with parkinsonian tremor at the detected tremor frequency and reported an almost 50% reduction of the amplitude of tremor. Other authors have speculated that tACS may help with the consolidation of motor learning in patients with PD (Broeder et al. 2023). These are obviously early days for the use of tACS in patients with PD, but further study may be warranted to optimize tACS procedures for more long-term benefits.

Other technologies have also been explored as potential treatment of PD. For example, a recent study tested whether transcranial ultrasonic stimulation could be used as an antiparkinsonian modality, due to its ability to modulate cortical excitability. These authors reported that the ultrasonic treatment resulted in decreased beta-band activity and a mild reduction of beta-gamma PAC (Wang et al. 2020).

Consistent with the reported changes of cortical GABAergic circuits, several studies found increased cognitive and motor function of PD patients following the administration of hypnotic drugs (e.g. zolpidem, a GABA-A receptor modulator) (Chen et al. 2008; Huang et al. 2012), perhaps by rebalancing M1 beta band activities between two hemispheres (Hall et al. 2014).

## Conclusion

While there is no doubt that the motor cortices are involved in the pathophysiology of parkinsonism, the details of this involvement are just emerging, producing a bewildering array of sometimes incongruent facts from different experimental domains.

Traditional models of activity abnormalities in the basal ganglia–thalamocortical circuitry have suggested that parkinsonism is triggered by dopamine loss in the basal ganglia and that the pathologic subcortical signals act to disrupt the otherwise intact cortical processing in areas such as the SMA and M1. There is certainly support for the notion that subcortical abnormalities impact cortical activities in the predicted manner and that subcortical interventions in patients with PD (such as DBS) work, in part, by allowing cerebral cortex to function more normally. However, this is clearly not the whole story. In fact, considerable evidence suggests that the motor cortices are an early target of primary and secondary pathology in PD, ranging from monoaminergic and glutamatergic denervation to neuroplastic synaptic and cellular changes. Recognition of these cortical abnormalities that are not necessarily correctable with conventional antiparkinsonian treatments, and further understanding of the anatomical and functional heterogeneity of cortical neurons and their pathophysiological changes during the development of parkinsonism, may present us with new therapeutic opportunities to help patients with PD to overcome their motor and nonmotor symptoms.

## References

[ref1] Abbasi O , Hirschmann J, Storzer L, Ozkurt TE, Elben S, Vesper J, Wojtecki L, Schmitz G, Schnitzler A, Butz M. Unilateral deep brain stimulation suppresses alpha and beta oscillations in sensorimotor cortices. NeuroImage. 2018:174:201–207. 10.1016/j.neuroimage.2018.03.026. 29551459

[ref2] Albin RL , Young AB, Penney JB. The functional anatomy of basal ganglia disorders. Trends Neurosci. 1989:12(10):366–375. 10.1016/0166-2236(89)90074-X. 2479133

[ref3] Albin RL , Koeppe RA, Bohnen NI, Wernette K, Kilbourn MA, Frey KA. Spared caudal brainstem SERT binding in early Parkinson's disease. J Cereb Blood Flow Metab. 2008:28(3):441–444. 10.1038/sj.jcbfm.9600599. 18073772

[ref4] Albin RL , van der Zee S, van Laar T, Sarter M, Lustig C, Muller M, Bohnen NI. Cholinergic systems, attentional-motor integration, and cognitive control in Parkinson's disease. Prog Brain Res. 2022:269:345–371. 10.1016/bs.pbr.2022.01.011. 35248201 PMC8957710

[ref5] Andersen KB , Hansen AK, Sommerauer M, Fedorova TD, Knudsen K, Vang K, Van Den Berge N, Kinnerup M, Nahimi A, Pavese N, et al. Altered sensorimotor cortex noradrenergic function in idiopathic REM sleep behaviour disorder - a PET study. Parkinsonism Relat Disord. 2020:75:63–69. 10.1016/j.parkreldis.2020.05.013. 32480309

[ref6] Azmitia EC , Nixon R. Dystrophic serotonergic axons in neurodegenerative diseases. Brain Res. 2008:1217:185–194. 10.1016/j.brainres.2008.03.060. 18502405 PMC3405553

[ref7] Ballanger B , Beaudoin-Gobert M, Neumane S, Epinat J, Metereau E, Duperrier S, Broussolle E, Thobois S, Bonnefoi F, Tourvielle C, et al. Imaging dopamine and serotonin systems on MPTP monkeys: a longitudinal PET investigation of compensatory mechanisms. J Neurosci. 2016:36(5):1577–1589. 10.1523/JNEUROSCI.2010-15.2016. 26843639 PMC6601989

[ref8] Baudrexel S , Witte T, Seifried C, von Wegner F, Beissner F, Klein JC, Steinmetz H, Deichmann R, Roeper J, Hilker R. Resting state fMRI reveals increased subthalamic nucleus–motor cortex connectivity in Parkinson's disease. NeuroImage. 2011:55(4):1728–1738. 10.1016/j.neuroimage.2011.01.017. 21255661

[ref9] Beaudoin-Gobert M , Epinat J, Metereau E, Duperrier S, Neumane S, Ballanger B, Lavenne F, Liger F, Tourvielle C, Bonnefoi F, et al. Behavioural impact of a double dopaminergic and serotonergic lesion in the non-human primate. Brain. 2015:138(9):2632–2647. 10.1093/brain/awv183. 26117365

[ref10] Beier KT , Steinberg EE, DeLoach KE, Xie S, Miyamichi K, Schwarz L, Gao XJ, Kremer EJ, Malenka RC, Luo L. Circuit architecture of VTA dopamine neurons revealed by systematic input-output mapping. Cell. 2015:162(3):622–634. 10.1016/j.cell.2015.07.015. 26232228 PMC4522312

[ref11] Berardelli A , Inghilleri M, Rothwell JC, Romeo S, Curra A, Gilio F, Modugno N, Manfredi M. Facilitation of muscle evoked responses after repetitive cortical stimulation in man. Exp Brain Res. 1998:122(1):79–84. 10.1007/s002210050493. 9772114

[ref12] Berger B , Trottier S, Verney C, Gaspar P, Alvarez C. Regional and laminar distribution of the dopamine and serotonin innervation in the macaque cerebral cortex: a radioautographic study. J Comp Neurol. 1988:273(1):99–119. 10.1002/cne.902730109. 3209731

[ref13] Berger B , Gaspar P, Verney C. Dopaminergic innervation of the cerebral cortex: unexpected differences between rodents and primates. Trends Neurosci. 1991:14(1):21–27. 10.1016/0166-2236(91)90179-X. 1709528

[ref14] Bergman H , Feingold A, Nini A, Raz A, Slovin H, Abeles M, Vaadia E. Physiological aspects of information processing in the basal ganglia of normal and parkinsonian primates. Trends Neurosci. 1998:21(1):32–38. 10.1016/S0166-2236(97)01151-X. 9464684

[ref15] Blesa J , Foffani G, Dehay B, Bezard E, Obeso JA. Motor and non-motor circuit disturbances in early Parkinson disease: which happens first? Nat Rev Neurosci. 2022:23(2):115–128. 10.1038/s41583-021-00542-9. 34907352

[ref16] Bock A , Kuhn AA, Trahms L, Sander TH. Validity of subthalamic-cortical coherency observed in patients with Parkinson's disease. Biomed Tech (Berl). 2013:58(2):157–164. 10.1515/bmt-2012-0023. 23446923

[ref17] Bočková M , Rektor I. Impairment of brain functions in Parkinson’s disease reflected by alterations in neural connectivity in EEG studies: a viewpoint. Clin Neurophysiol. 2019:130(2):239–247. 10.1016/j.clinph.2018.11.013. 30580247

[ref18] Boeve BF . Idiopathic REM sleep behaviour disorder in the development of Parkinson's disease. Lancet Neurol. 2013:12(5):469–482. 10.1016/S1474-4422(13)70054-1. 23578773 PMC4779953

[ref19] Bohnen NI , van der Zee S, Albin R. Cholinergic centro-cingulate network in Parkinson disease and normal aging. Aging (Albany NY). 2023:15(20):10817–10820. 10.18632/aging.205209. 37899134 PMC10637805

[ref20] Bologna M , Suppa A, Conte A, Latorre A, Rothwell JC, Berardelli A. Are studies of motor cortex plasticity relevant in human patients with Parkinson’s disease? Clin Neurophysiol. 2016:127(1):50–59. 10.1016/j.clinph.2015.02.009. 25792075

[ref21] Bonaiuto JJ , Little S, Neymotin SA, Jones SR, Barnes GR, Bestmann S. Laminar dynamics of high amplitude beta bursts in human motor cortex. NeuroImage. 2021:242:118479. 10.1016/j.neuroimage.2021.118479. 34407440 PMC8463839

[ref22] Boon LI , Hillebrand A, Potters WV, de Bie RMA, Prent N, Bot M, Schuurman PR, Stam CJ, van Rootselaar AF, Berendse HW. Motor effects of deep brain stimulation correlate with increased functional connectivity in Parkinson's disease: An MEG study. Neuroimage Clin. 2020:26:102225. 10.1016/j.nicl.2020.102225. 32120294 PMC7049661

[ref23] Boon LI , Potters WV, Hillebrand A, de Bie RMA, Bot M, Richard Schuurman P, van den Munckhof P, Twisk JW, Stam CJ, Berendse HW, et al. Magnetoencephalography to measure the effect of contact point-specific deep brain stimulation in Parkinson's disease: a proof of concept study. Neuroimage Clin. 2023:38:103431. 10.1016/j.nicl.2023.103431. 37187041 PMC10197095

[ref24] Boylan LS , Pullman SL, Lisanby SH, Spicknall KE, Sackeim HA. Repetitive transcranial magnetic stimulation to SMA worsens complex movements in Parkinson's disease. Clin Neurophysiol. 2001:112(2):259–264. 10.1016/S1388-2457(00)00519-8. 11165527

[ref25] Braak H , Del Tredici K. Neuroanatomy and pathology of sporadic Parkinson's disease. Adv Anat Embryol Cell Biol. 2009:201:1–119. 19230552

[ref26] Braak H , Del Tredici K, Rub U, de Vos RA, Jansen Steur EN, Braak E. Staging of brain pathology related to sporadic Parkinson's disease. Neurobiol Aging. 2003:24(2):197–211. 10.1016/S0197-4580(02)00065-9. 12498954

[ref27] Braak H , Ghebremedhin E, Rüb U, Bratzke H, Del Tredici K. Stages in the development of Parkinson's disease-related pathology. Cell Tissue Res. 2004:318(1):121–134. 10.1007/s00441-004-0956-9. 15338272

[ref28] Braak H , de Vos RA, Bohl J, Del Tredici K. Gastric alpha-synuclein immunoreactive inclusions in Meissner's and Auerbach's plexuses in cases staged for Parkinson's disease-related brain pathology. Neurosci Lett. 2006a:396(1):67–72. 10.1016/j.neulet.2005.11.012. 16330147

[ref29] Braak H , Rub U, Schultz C, Del Tredici K. Vulnerability of cortical neurons to Alzheimer's and Parkinson's diseases. J Alzheimers Dis. 2006b:9(s3):35–44. 10.3233/JAD-2006-9S305. 16914843

[ref30] Brazhnik E , Cruz AV, Avila I, Wahba MI, Novikov N, Ilieva NM, McCoy AJ, Gerber C, Walters JR. State-dependent spike and local field synchronization between motor cortex and substantia Nigra in Hemiparkinsonian rats. J Neurosci. 2012:32(23):7869–7880. 10.1523/JNEUROSCI.0943-12.2012. 22674263 PMC3423905

[ref31] Brazhnik E , McCoy AJ, Novikov N, Hatch CE, Walters JR. Ventral medial thalamic nucleus promotes synchronization of increased high Beta oscillatory activity in the basal ganglia–Thalamocortical network of the Hemiparkinsonian rat. J Neurosci. 2016:36(15):4196–4208. 10.1523/JNEUROSCI.3582-15.2016. 27076419 PMC4829645

[ref32] Brazhnik E , Novikov N, McCoy AJ, Ilieva NM, Ghraib MW, Walters JR. Early decreases in cortical mid-gamma peaks coincide with the onset of motor deficits and precede exaggerated beta build-up in rat models for Parkinson's disease. Neurobiol Dis. 2021:155:105393. 10.1016/j.nbd.2021.105393. 34000417 PMC8422282

[ref33] Brittain JS , Probert-Smith P, Aziz TZ, Brown P. Tremor suppression by rhythmic transcranial current stimulation. Curr Biol. 2013:23(5):436–440. 10.1016/j.cub.2013.01.068. 23416101 PMC3629558

[ref34] Broeder S , Vandendoorent B, Hermans P, Nackaerts E, Verheyden G, Meesen R, de Xivry JO, Nieuwboer A. Transcranial direct current stimulation enhances motor learning in Parkinson's disease: a randomized controlled trial. J Neurol. 2023:270(7):3442–3450. 10.1007/s00415-023-11669-3. 36952012 PMC10035486

[ref35] Brooks DJ , Piccini P. Imaging in Parkinson's disease: the role of monoamines in behavior. Biol Psychiatry. 2006:59(10):908–918. 10.1016/j.biopsych.2005.12.017. 16581032

[ref36] Brown P , Marsden CD. Bradykinesia and impairment of EEG desynchronization in Parkinson's disease. Mov Disord. 1999:14(3):423–429. 10.1002/1531-8257(199905)14:3<423::AID-MDS1006>3.0.CO;2-V. 10348464

[ref37] Brys M , Fox MD, Agarwal S, Biagioni M, Dacpano G, Kumar P, Pirraglia E, Chen R, Wu A, Fernandez H, et al. Multifocal repetitive TMS for motor and mood symptoms of Parkinson disease: a randomized trial. Neurology. 2016:87(18):1907–1915. 10.1212/WNL.0000000000003279. 27708129 PMC5100715

[ref38] Buddhala C , Loftin SK, Kuley BM, Cairns NJ, Campbell MC, Perlmutter JS, Kotzbauer PT. Dopaminergic, serotonergic, and noradrenergic deficits in Parkinson disease. Ann Clin Transl Neurol. 2015:2(10):949–959. 10.1002/acn3.246. 26478895 PMC4603378

[ref39] Burciu RG , Ofori E, Shukla P, Planetta PJ, Snyder AF, Li H, Hass CJ, Okun MS, McFarland NR, Vaillancourt DE. Distinct patterns of brain activity in progressive supranuclear palsy and Parkinson's disease. Mov Disord. 2015:30(9):1248–1258. 10.1002/mds.26294. 26148135 PMC4578977

[ref40] Camps M , Kelly PH, Palacios JM. Autoradiographic localization of dopamine D 1 and D 2 receptors in the brain of several mammalian species. J Neural Transm Gen Sect. 1990:80(2):105–127. 10.1007/BF01257077. 2138461

[ref41] Candy JM , Perry EK, Perry RH, Court JA, Oakley AE, Edwardson JA. The current status of the cortical cholinergic system in Alzheimer's disease and Parkinson's disease. Prog Brain Res. 1986:70:105–132. 10.1016/S0079-6123(08)64300-9. 3554348

[ref42] Cao C , Li D, Jiang T, Ince NF, Zhan S, Zhang J, Sha Z, Sun B. Resting state cortical oscillations of patients with Parkinson disease and with and without subthalamic deep brain stimulation: a magnetoencephalography study. J Clin Neurophysiol. 2015:32(2):109–118. 10.1097/WNP.0000000000000137. 25233246

[ref43] Cao CY , Zeng K, Li DY, Zhan SK, Li XL, Sun BM. Modulations on cortical oscillations by subthalamic deep brain stimulation in patients with Parkinson disease: a MEG study. Neurosci Lett. 2017:636:95–100. 10.1016/j.neulet.2016.11.009. 27818350

[ref44] Cao C , Li D, Zhan S, Zhang C, Sun B, Litvak V. L-dopa treatment increases oscillatory power in the motor cortex of Parkinson's disease patients. Neuroimage Clin. 2020:26:102255. 10.1016/j.nicl.2020.102255. 32361482 PMC7195547

[ref45] Caspers J , Rubbert C, Eickhoff SB, Hoffstaedter F, Südmeyer M, Hartmann CJ, Sigl B, Teichert N, Aissa J, Turowski B, et al. Within- and across-network alterations of the sensorimotor network in Parkinson’s disease. Neuroradiology. 2021:63(12):2073–2085. 10.1007/s00234-021-02731-w. 34019112 PMC8589810

[ref46] Chen R , Kumar S, Garg RR, Lang AE. Impairment of motor cortex activation and deactivation in Parkinson's disease. Clin Neurophysiol. 2001:112(4):600–607. 10.1016/S1388-2457(01)00466-7. 11275531

[ref47] Chen YY , Sy HN, Wu SL. Zolpidem improves akinesia, dystonia and dyskinesia in advanced Parkinson's disease. J Clin Neurosci. 2008:15(8):955–956. 10.1016/j.jocn.2007.07.082. 18485713

[ref48] Chen L , Daniels S, Kim Y, Chu HY. Cell type-specific decrease of the intrinsic excitability of motor cortical pyramidal neurons in parkinsonism. J Neurosci. 2021:41(25):5553–5565. 10.1523/JNEUROSCI.2694-20.2021. 34006589 PMC8221604

[ref49] Chen L , Daniels S, Dvorak R, Chu HY. Reduced thalamic excitation to motor cortical pyramidal tract neurons in parkinsonism. Sci Adv. 2023:9(34):eadg3038. 10.1126/sciadv.adg3038. 37611096 PMC10446482

[ref50] Cherian S , Simms G, Chen L, Chu HY. Loss of midbrain dopamine neurons does not Alter GABAergic inhibition mediated by Parvalbumin-expressing interneurons in mouse primary motor cortex. Eneuro. 2024:11(5):ENEURO.0010–ENEU24.2024. 10.1523/ENEURO.0010-24.2024. 38658137 PMC11082919

[ref51] Chu J , Wagle-Shukla A, Gunraj C, Lang AE, Chen R. Impaired presynaptic inhibition in the motor cortex in Parkinson disease. Neurology. 2009:72(9):842–849. 10.1212/01.wnl.0000343881.27524.e8. 19255412

[ref52] Chu H-Y , McIver EL, Kovaleski RF, Atherton JF, Bevan MD. Loss of hyperdirect pathway cortico-subthalamic inputs following degeneration of midbrain dopamine neurons. Neuron. 2017:95(6):1306–1318.e5. 10.1016/j.neuron.2017.08.038. 28910619 PMC5679443

[ref53] Chu C , Liu S, He N, Zeng Z, Wang J, Zhang Z, Zeljic K, Ovd S, Sun B, Yan F, et al. Subthalamic stimulation modulates motor network in Parkinson’s disease: recover, relieve and remodel. Brain. 2023:146:5. 10.1093/brain/awad004 36623929

[ref54] Chung JW , Burciu RG, Ofori E, Coombes SA, Christou EA, Okun MS, Hess CW, Vaillancourt DE. Beta-band oscillations in the supplementary motor cortex are modulated by levodopa and associated with functional activity in the basal ganglia. NeuroImage Clin. 2018:19:559–571. 10.1016/j.nicl.2018.05.021. 29984164 PMC6029579

[ref55] Conti M , Stefani A, Bovenzi R, Cerroni R, Garasto E, Placidi F, Liguori C, Schirinzi T, Mercuri NB, Pierantozzi M. STN-DBS induces acute changes in β-band cortical functional connectivity in patients with Parkinson’s disease. Brain Sci. 2022:12(12):1606. 10.3390/brainsci12121606. 36552066 PMC9775160

[ref56] Cousineau J , Lescouzères L, Taupignon A, Delgado-Zabalza L, Valjent E, Baufreton J, Bon-Jégo ML. Dopamine D2-like receptors modulate intrinsic properties and synaptic transmission of Parvalbumin interneurons in the mouse primary motor cortex. Eneuro. 2020:7(3):ENEURO.0081–ENEU20.2020. 10.1523/ENEURO.0081-20.2020. PMC7240291 32321772

[ref57] Cousineau J , Plateau V, Baufreton J, Bon-Jégo ML. Dopaminergic modulation of primary motor cortex: from cellular and synaptic mechanisms underlying motor learning to cognitive symptoms in Parkinson’s disease. Neurobiol Dis. 2022:167:105674. 10.1016/j.nbd.2022.105674. 35245676

[ref58] Cross KA , Malekmohammadi M, Woo Choi J, Pouratian N. Movement-related changes in pallidocortical synchrony differentiate action execution and observation in humans. Clin Neurophysiol. 2021:132(8):1990–2001. 10.1016/j.clinph.2021.03.037. 33980469 PMC8286311

[ref59] Crowell AL , Ryapolova-Webb ES, Ostrem JL, Galifianakis NB, Shimamoto S, Lim DA, Starr PA. Oscillations in sensorimotor cortex in movement disorders: an electrocorticography study. Brain. 2012:135(2):615–630. 10.1093/brain/awr332. 22252995 PMC3281473

[ref60] Day M , Wang Z, Ding J, An X, Ingham CA, Shering AF, Wokosin D, Ilijic E, Sun Z, Sampson AR, et al. Selective elimination of glutamatergic synapses on striatopallidal neurons in Parkinson disease models. Nat Neurosci. 2006:9(2):251–259. 10.1038/nn1632. 16415865

[ref123] de Hemptinne C , Ryapolova-Webb ES, Air EL, Garcia PA, Miller KJ, Ojemann JG, Ostrem JL, Galifianakis NB, Starr PA. Exaggerated phase-amplitude coupling in the primary motor cortex in Parkinson disease. Proc Natl Acad Sci U S A. 2013:110(12):4780–4785. 10.1073/pnas.1214546110. 23471992 PMC3606991

[ref124] de Hemptinne C , Swann NC, Ostrem JL, Ryapolova-Webb ES, San Luciano M, Galifianakis NB, Starr PA. Therapeutic deep brain stimulation reduces cortical phase-amplitude coupling in Parkinson's disease. Nat Neurosci. 2015:18(5):779–786. 10.1038/nn.3997. 25867121 PMC4414895

[ref61] Defebvre L , Bourriez JL, Derambure P, Duhamel A, Guieu JD, Destee A. Influence of chronic administration of L-DOPA on event-related desynchronization of mu rhythm preceding voluntary movement in Parkinson's disease. Electroencephalogr Clin Neurophysiol. 1998:109(2):161–167. 10.1016/S0924-980X(97)00085-4. 9741807

[ref62] Dejean C , Hyland B, Arbuthnott G. Cortical effects of subthalamic stimulation correlate with behavioral recovery from dopamine antagonist induced akinesia. Cereb Cortex. 2009:19(5):1055–1063. 10.1093/cercor/bhn149. 18787234

[ref63] Del Tredici K , Braak H. Review: sporadic Parkinson's disease: development and distribution of alpha-synuclein pathology. Neuropathol Appl Neurobiol. 2016:42(1):33–50. 10.1111/nan.12298. 26662475

[ref64] DeLong MR . Primate models of movement disorders of basal ganglia origin. Trends Neurosci. 1990:13(7):281–285. 10.1016/0166-2236(90)90110-V. 1695404

[ref65] Devergnas A , Wichmann T. Cortical potentials evoked by deep brain stimulation in the subthalamic area. Front Syst Neurosci. 2011:5:30. 10.3389/fnsys.2011.00030. 21625611 PMC3097379

[ref66] Devergnas A , Pittard D, Bliwise D, Wichmann T. Relationship between oscillatory activity in the cortico-basal ganglia network and parkinsonism in MPTP-treated monkeys. Neurobiol Dis. 2014:68:156–166. 10.1016/j.nbd.2014.04.004. 24768805 PMC4275129

[ref67] Devos D , Labyt E, Derambure P, Bourriez JL, Cassim F, Guieu JD, Destee A, Defebvre L. Effect of L-Dopa on the pattern of movement-related (de)synchronisation in advanced Parkinson's disease. Neurophysiol Clin. 2003:33(5):203–212. 10.1016/j.neucli.2003.10.001. 14672820

[ref68] Dickson DW , Schmidt ML, Lee VM, Zhao ML, Yen SH, Trojanowski JQ. Immunoreactivity profile of hippocampal CA2/3 neurites in diffuse Lewy body disease. Acta Neuropathol. 1994:87(3):269–276. 10.1007/BF00296742. 7912027

[ref69] Dickson DW , Uchikado H, Fujishiro H, Tsuboi Y. Evidence in favor of Braak staging of Parkinson's disease. Mov Disord. 2010:25(Suppl 1):S78–S82. 10.1002/mds.22637. 20187227

[ref70] Disbrow EA , Glassy ND, Dressler EM, Russo K, Franz EA, Turner RS, Ventura MI, Hinkley L, Zweig R, Nagarajan SS, et al. Cortical oscillatory dysfunction in Parkinson disease during movement activation and inhibition. PLoS One. 2022:17(3):e0257711. 10.1371/journal.pone.0257711. 35245294 PMC8896690

[ref71] Doherty DW , Chen L, Smith Y, Wichmann T, H-y C, Lytton WW. Decreased cellular excitability of pyramidal tract neurons in primary motor cortex leads to paradoxically increased network activity in simulated parkinsonian motor cortex. Biorxiv. 2024: 2024.2005.2023.595566. 10.1101/2024.05.23.595566.

[ref72] Doudet DJ , Gross C, Arluison M, Bioulac B. Modifications of precentral cortex discharge and EMG activity in monkeys with MPTP-induced lesions of DA nigral neurons. Exp Brain Res. 1990:80(1):177–188. 10.1007/BF00228859. 1972680

[ref73] Drouot X , Oshino S, Jarraya B, Besret L, Kishima H, Remy P, Dauguet J, Lefaucheur JP, Dollé F, Condé F, et al. Functional recovery in a primate model of Parkinson's disease following motor cortex stimulation. Neuron. 2004:44(5):769–778. 10.1016/j.neuron.2004.11.023. 15572109

[ref74] Dum RP , Strick PL. Motor areas in the frontal lobe of the primate. Physiol Behav. 2002:77(4–5):677–682. 10.1016/S0031-9384(02)00929-0. 12527018

[ref75] Eguchi K , Shirai S, Matsushima M, Kano T, Ichikawa T, Yamazaki K, Hamauchi S, Sasamori T, Seki T, Kitagawa M, et al. Chronic deep brain stimulation reduces cortical β-γ phase amplitude-coupling in patients with Parkinson's disease. Parkinsonism Relat Disord. 2021:89:148–150. 10.1016/j.parkreldis.2021.07.017. 34303200

[ref76] Elahi B , Elahi B, Chen R. Effect of transcranial magnetic stimulation on Parkinson motor function—systematic review of controlled clinical trials. Mov Disord. 2009:24(3):357–363. 10.1002/mds.22364. 18972549

[ref77] Elsworth JD , Deutch AY, Redmond DE Jr, Sladek JR Jr, Roth RH. MPTP-induced parkinsonism: relative changes in dopamine concentration in subregions of substantia nigra, ventral tegmental area and retrorubral field of symptomatic and asymptomatic vervet monkeys. Brain Res. 1990:513(2):320–324. 10.1016/0006-8993(90)90474-P. 2350702

[ref78] Elsworth JD , Leranth C, Redmond DE Jr, Roth RH. Loss of asymmetric spine synapses in prefrontal cortex of motor-asymptomatic, dopamine-depleted, cognitively impaired MPTP-treated monkeys. Int J Neuropsychopharmacol. 2013:16(4):905–912. 10.1017/S1461145712000892. 22947206 PMC3733504

[ref79] Engeln M , De Deurwaerdere P, Li Q, Bezard E, Fernagut PO. Widespread monoaminergic dysregulation of both motor and non-motor circuits in parkinsonism and dyskinesia. Cereb Cortex. 2015:25(9):2783–2792. 10.1093/cercor/bhu076. 24770706

[ref80] Evangelisti S , Pittau F, Testa C, Rizzo G, Gramegna LL, Ferri L, Coito A, Cortelli P, Calandra-Buonaura G, Bisquoli F, et al. L-Dopa modulation of brain connectivity in Parkinson’s disease patients: a pilot EEG-fMRI study. Front Neurosci. 2019:13:611. 10.3389/fnins.2019.00611. 31258465 PMC6587436

[ref81] Feigin A , Fukuda M, Dhawan V, Przedborski S, Jackson-Lewis V, Mentis MJ, Moeller JR, Eidelberg D. Metabolic correlates of levodopa response in Parkinson’s disease. Neurol J Am Hear Assoc. 2001:57(11):2083–2088. 10.1212/WNL.57.11.2083. 11739830

[ref82] Feingold J , Gibson DJ, DePasquale B, Graybiel AM. Bursts of beta oscillation differentiate postperformance activity in the striatum and motor cortex of monkeys performing movement tasks. Proc Natl Acad Sci. 2015:112(44):13687–13692. 10.1073/pnas.1517629112. 26460033 PMC4640760

[ref83] Fieblinger T , Graves SM, Sebel LE, Alcacer C, Plotkin JL, Gertler TS, Chan CS, Heiman M, Greengard P, Cenci MA, et al. Cell type-specific plasticity of striatal projection neurons in parkinsonism and L-DOPA-induced dyskinesia. Nat Commun. 2014:5(1):5316. 10.1038/ncomms6316. 25360704 PMC4431763

[ref84] Fregni F , Simon DK, Wu A, Pascual-Leone A. Non-invasive brain stimulation for Parkinson's disease: a systematic review and meta-analysis of the literature. J Neurol Neurosurg Psychiatry. 2005:76(12):1614–1623. 10.1136/jnnp.2005.069849. 16291882 PMC1739437

[ref85] Fu Y , Zhou L, Li H, Hsiao JT, Li B, Tanglay O, Auwyang AD, Wang E, Feng J, Kim WS, et al. Adaptive structural changes in the motor cortex and white matter in Parkinson's disease. Acta Neuropathol. 2022:144(5):861–879. 10.1007/s00401-022-02488-3. 36053316 PMC9547807

[ref86] Galvan A , Wichmann T. Pathophysiology of parkinsonism. Clin Neurophysiol. 2008:119(7):1459–1474. 10.1016/j.clinph.2008.03.017. 18467168 PMC2467461

[ref87] Gaspar P , Berger B, Febvret A, Vigny A, Henry JP. Catecholamine innervation of the human cerebral cortex as revealed by comparative immunohistochemistry of tyrosine hydroxylase and dopamine-beta-hydroxylase. J Comp Neurol. 1989:279(2):249–271. 10.1002/cne.902790208. 2563268

[ref88] Gaspar P , Duyckaerts C, Alvarez C, Javoy-Agid F, Berger B. Alterations of dopaminergic and noradrenergic innervations in motor cortex in Parkinson's disease. Ann Neurol. 1991:30(3):365–374. 10.1002/ana.410300308. 1683212

[ref89] Gaspar P , Stepniewska I, Kaas JH. Topography and collateralization of the dopaminergic projections to motor and lateral prefrontal cortex in owl monkeys. J Comp Neurol. 1992:325(1):1–21. 10.1002/cne.903250102. 1362430

[ref90] Gaspar P , Bloch B, Le Moine C. D1 and D2 receptor gene expression in the rat frontal cortex: cellular localization in different classes of efferent neurons. Eur J Neurosci. 1995:7(5):1050–1063. 10.1111/j.1460-9568.1995.tb01092.x. 7613610

[ref91] Gatev P , Wichmann T. Interactions between cortical rhythms and spiking activity of single basal ganglia neurons in the normal and parkinsonian state. Cereb Cortex. 2009:19(6):1330–1344. 10.1093/cercor/bhn171. 18842667 PMC2677649

[ref92] Geerts H , Bergeler S, Lytton WW, van der Graaf PH. Computational neurosciences and quantitative systems pharmacology: a powerful combination for supporting drug development in neurodegenerative diseases. J Pharmacokinet Pharmacodyn. 2023. 10.1007/s10928-023-09876-6. 37505397

[ref93] Gilio F , Curra A, Inghilleri M, Lorenzano C, Manfredi M, Berardelli A. Repetitive magnetic stimulation of cortical motor areas in Parkinson's disease: implications for the pathophysiology of cortical function. Mov Disord. 2002:17(3):467–473. 10.1002/mds.1255. 12112192

[ref94] Goedert M , Spillantini MG, Del Tredici K, Braak H. 100 years of Lewy pathology. Nat Rev Neurol. 2012:9(1):13–24. 10.1038/nrneurol.2012.242. 23183883

[ref95] Goldberg JA , Boraud T, Maraton S, Haber SN, Vaadia E, Bergman H. Enhanced synchrony among primary motor cortex neurons in the 1-Methyl-4-Phenyl-1,2,3,6-Tetrahydropyridine primate model of Parkinson's disease. J Neurosci. 2002:22(11):4639–4653. 10.1523/JNEUROSCI.22-11-04639.2002. 12040070 PMC6758785

[ref96] Grafton ST . Contributions of functional imaging to understanding parkinsonian symptoms. Curr Opin Neurobiol. 2004:14(6):715–719. 10.1016/j.conb.2004.10.010. 15582373

[ref97] Grafton ST , DeLong M. Tracing the brain's circuitry with functional imaging. Nat Med. 1997:3(6):602–603. 10.1038/nm0697-602. 9176479

[ref98] Grafton ST , Volz LJ. From ideas to action: the prefrontal-premotor connections that shape motor behavior. Handb Clin Neurol. 2019:163:237–255. 10.1016/B978-0-12-804281-6.00013-6. 31590733

[ref99] Grafton ST , Sutton J, Couldwell W, Lew M, Waters C. Network analysis of motor system connectivity in Parkinson's disease: modulation of thalamocortical interactions after pallidotomy. Hum Brain Mapp. 2004:2(1–2):45–55. 10.1002/hbm.460020106.

[ref100] Grafton ST , Turner RS, Desmurget M, Bakay R, Delong M, Vitek J, Crutcher M. Normalizing motor-related brain activity: subthalamic nucleus stimulation in Parkinson disease. Neurology. 2006:66(8):1192–1199. 10.1212/01.wnl.0000214237.58321.c3. 16636237

[ref101] Gu S-C , Ye Q, Yuan C-X. Metabolic pattern analysis of 18F-FDG PET as a marker for Parkinson’s disease: a systematic review and meta-analysis. Rev Neurosci. 2019:30(7):743–756. 10.1515/revneuro-2018-0061. 31050657

[ref102] Guerra A , Asci F, D'Onofrio V, Sveva V, Bologna M, Fabbrini G, Berardelli A, Suppa A. Enhancing gamma oscillations restores primary motor cortex plasticity in Parkinson's disease. J Neurosci. 2020:40(24):4788–4796. 10.1523/JNEUROSCI.0357-20.2020. 32430296 PMC7294804

[ref103] Guerra A , D'Onofrio V, Asci F, Ferreri F, Fabbrini G, Berardelli A, Bologna M. Assessing the interaction between L-dopa and gamma-transcranial alternating current stimulation effects on primary motor cortex plasticity in Parkinson's disease. Eur J Neurosci. 2023:57(1):201–212. 10.1111/ejn.15867. 36382537 PMC10100043

[ref104] Guo L , Xiong H, Kim J-I, Wu Y-W, Lalchandani RR, Cui Y, Shu Y, Xu T, Ding JB. Dynamic rewiring of neural circuits in the motor cortex in mouse models of Parkinson's disease. Nat Neurosci. 2015:18(9):1299–1309. 10.1038/nn.4082. 26237365 PMC4551606

[ref105] Guo K , Yamawaki N, Svoboda K, Shepherd GMG. Anterolateral motor cortex connects with a medial subdivision of ventromedial thalamus through cell type-specific circuits, forming an excitatory Thalamo-Cortico-thalamic loop via layer 1 apical tuft dendrites of layer 5B pyramidal tract type neurons. J Neurosci. 2018:38(41):8787–8797. 10.1523/JNEUROSCI.1333-18.2018. 30143573 PMC6181310

[ref106] Gutierrez JC , Seijo FJ, Alvarez Vega MA, Fernandez Gonzalez F, Lozano Aragoneses B, Blazquez M. Therapeutic extradural cortical stimulation for Parkinson's disease: report of six cases and review of the literature. Clin Neurol Neurosurg. 2009:111(8):703–707. 10.1016/j.clineuro.2009.06.006. 19604625

[ref107] Guttman M , Boileau I, Warsh J, Saint-Cyr JA, Ginovart N, McCluskey T, Houle S, Wilson A, Mundo E, Rusjan P, et al. Brain serotonin transporter binding in non-depressed patients with Parkinson's disease. Eur J Neurol. 2007:14(5):523–528. 10.1111/j.1468-1331.2007.01727.x. 17437611

[ref108] Hall SD , Prokic EJ, McAllister CJ, Ronnqvist KC, Williams AC, Yamawaki N, Witton C, Woodhall GL, Stanford IM. GABA-mediated changes in inter-hemispheric beta frequency activity in early-stage Parkinson's disease. Neuroscience. 2014:281:68–76. 10.1016/j.neuroscience.2014.09.037. 25261686 PMC4222199

[ref109] Halliday GM . Thalamic changes in Parkinson's disease. Parkinsonism Relat Disord. 2009:15(Suppl 3):S152–S155. 10.1016/S1353-8020(09)70804-1. 20082979

[ref110] Halliday GM , Blumbergs PC, Cotton RG, Blessing WW, Geffen LB. Loss of brainstem serotonin- and substance P-containing neurons in Parkinson's disease. Brain Res. 1990a:510(1):104–107. 10.1016/0006-8993(90)90733-R. 1691042

[ref111] Halliday GM , Li YW, Blumbergs PC, Joh TH, Cotton RG, Howe PR, Blessing WW, Geffen LB. Neuropathology of immunohistochemically identified brainstem neurons in Parkinson's disease. Ann Neurol. 1990b:27(4):373–385. 10.1002/ana.410270405. 1972319

[ref112] Halliday GM , Macdonald V, Henderson JM. A comparison of degeneration in motor thalamus and cortex between progressive supranuclear palsy and Parkinson's disease. Brain. 2005:128(10):2272–2280. 10.1093/brain/awh596. 16014651

[ref113] Halliday G , McCann H, Shepherd C. Evaluation of the Braak hypothesis: how far can it explain the pathogenesis of Parkinson's disease? Expert Rev Neurother. 2012:12(6):673–686. 10.1586/ern.12.47. 22650170

[ref114] Hamada M , Ugawa Y, Tsuji S, Effectiveness of rTms on Parkinson's Disease Study Group J. High-frequency rTMS over the supplementary motor area for treatment of Parkinson's disease. Mov Disord. 2008:23(11):1524–1531. 10.1002/mds.22168. 18548577

[ref115] Hammond C , Bergman H, Brown P. Pathological synchronization in Parkinson's disease: networks, models and treatments. Trends Neurosci. 2007:30(7):357–364. 10.1016/j.tins.2007.05.004. 17532060

[ref116] Hao L , Sheng Z, Ruijun W, Kun HZ, Peng Z, Yu H. Altered Granger causality connectivity within motor-related regions of patients with Parkinson’s disease: a resting-state fMRI study. Neuroradiology. 2020:62(1):63–69. 10.1007/s00234-019-02311-z. 31773188

[ref117] Haslinger B , Erhard P, Kämpfe N, Boecker H, Rummeny E, Schwaiger M, Conrad B, Ceballos-Baumann A. Event-related functional magnetic resonance imaging in Parkinson's disease before and after levodopa. Brain. 2001:124(3):558–570. 10.1093/brain/124.3.558. 11222456

[ref118] Haslinger B , Kalteis K, Boecker H, Alesch F, Ceballos-Baumann AO. Frequency-correlated decreases of motor cortex activity associated with subthalamic nucleus stimulation in Parkinson's disease. NeuroImage. 2005:28(3):598–606. 10.1016/j.neuroimage.2005.06.034. 16081302

[ref119] Heideman SG , Quinn AJ, Woolrich MW, van Ede F, Nobre AC. Dissecting beta-state changes during timed movement preparation in Parkinson's disease. Prog Neurobiol. 2020:184:101731. 10.1016/j.pneurobio.2019.101731. 31778771 PMC6977086

[ref120] Heinrichs-Graham E , Kurz MJ, Becker KM, Santamaria PM, Gendelman HE, Wilson TW. Hypersynchrony despite pathologically reduced beta oscillations in patients with Parkinson's disease: a pharmaco-magnetoencephalography study. J Neurophysiol. 2014a:112(7):1739–1747. 10.1152/jn.00383.2014. 25008416 PMC4157173

[ref121] Heinrichs-Graham E , Wilson TW, Santamaria PM, Heithoff SK, Torres-Russotto D, Hutter-Saunders JA, Estes KA, Meza JL, Mosley RL, Gendelman HE. Neuromagnetic evidence of abnormal movement-related beta desynchronization in Parkinson's disease. Cereb Cortex. 2014b:24(10):2669–2678. 10.1093/cercor/bht121. 23645717 PMC4153806

[ref122] Helmich RC , Derikx LC, Bakker M, Scheeringa R, Bloem BR, Toni I. Spatial remapping of cortico-striatal connectivity in Parkinson's disease. Cereb Cortex. 2010:20(5):1175–1186. 10.1093/cercor/bhp178. 19710357

[ref125] Henderson JM , Carpenter K, Cartwright H, Halliday GM. Degeneration of the Centre median-parafascicular complex in Parkinson's disease. Ann Neurol. 2000:47(3):345–352. 10.1002/1531-8249(200003)47:3<345::AID-ANA10>3.0.CO;2-V. 10716254

[ref126] Henderson JM , O'Sullivan DJ, Pell M, Fung VS, Hely MA, Morris JG, Halliday GM. Lesion of thalamic centromedian– parafascicular complex after chronic deep brain stimulation. Neurology. 2001:56(11):1576–1579. 10.1212/WNL.56.11.1576. 11402120

[ref127] Hershey T , Black K, Carl J, McGee-Minnich L, Snyder A, Perlmutter J. Long term treatment and disease severity change brain responses to levodopa in Parkinson’s disease. J Neurol Neurosurg Psychiatry. 2003:74(7):844–851. 10.1136/jnnp.74.7.844. 12810765 PMC1738560

[ref128] Hirschmann J , Ozkurt TE, Butz M, Homburger M, Elben S, Hartmann CJ, Vesper J, Wojtecki L, Schnitzler A. Distinct oscillatory STN-cortical loops revealed by simultaneous MEG and local field potential recordings in patients with Parkinson's disease. NeuroImage. 2011:55(3):1159–1168. 10.1016/j.neuroimage.2010.11.063. 21122819

[ref129] Hirschmann J , Ozkurt TE, Butz M, Homburger M, Elben S, Hartmann CJ, Vesper J, Wojtecki L, Schnitzler A. Differential modulation of STN-cortical and cortico-muscular coherence by movement and levodopa in Parkinson's disease. NeuroImage. 2013:68:203–213. 10.1016/j.neuroimage.2012.11.036. 23247184

[ref130] Horsager J , Okkels N, Hansen AK, Damholdt MF, Andersen KH, Fedorova TD, Munk OL, Danielsen EH, Pavese N, Brooks DJ, et al. Mapping cholinergic synaptic loss in Parkinson's disease: An [18F]FEOBV PET case-control study. J Parkinsons Dis. 2022:12(8):2493–2506. 10.3233/JPD-223489. 36336941

[ref131] Hosp JA , Luft AR. Dopaminergic meso-cortical projections to M1: role in motor learning and motor cortex plasticity. Front Neurol. 2013:4:145. 10.3389/fneur.2013.00145. 24109472 PMC3791680

[ref132] Hosp JA , Hertler B, Atiemo CO, Luft AR. Dopaminergic modulation of receptive fields in rat sensorimotor cortex. NeuroImage. 2011a:54(1):154–160. 10.1016/j.neuroimage.2010.07.029. 20643216

[ref133] Hosp JA , Pekanovic A, Rioult-Pedotti MS, Luft AR. Dopaminergic projections from midbrain to primary motor cortex mediate motor skill learning. J Neurosci. 2011b:31(7):2481–2487. 10.1523/JNEUROSCI.5411-10.2011. 21325515 PMC6623715

[ref134] Huang C , Tang C, Feigin A, Lesser M, Ma Y, Pourfar M, Dhawan V, Eidelberg D. Changes in network activity with the progression of Parkinson's disease. Brain. 2007:130(7):1834–1846. 10.1093/brain/awm086. 17470495 PMC4454378

[ref135] Huang HY , Hsu YT, Wu YC, Chiou SM, Kao CH, Tsai MC, Tsai CH. Zolpidem improves neuropsychiatric symptoms and motor dysfunction in a patient with Parkinson's disease after deep brain stimulation. Acta Neurol Taiwanica. 2012:21(2):84–86. 22879118

[ref136] Humphries MD , Obeso JA, Dreyer JK. Insights into Parkinson's disease from computational models of the basal ganglia. J Neurol Neurosurg Psychiatry. 2018:89(11):1181–1188. 10.1136/jnnp-2017-315922. 29666208 PMC6124639

[ref137] Hurtig HI , Trojanowski JQ, Galvin J, Ewbank D, Schmidt ML, Lee VM, Clark CM, Glosser G, Stern MB, Gollomp SM, et al. Alpha-synuclein cortical Lewy bodies correlate with dementia in Parkinson's disease. Neurology. 2000:54(10):1916–1921. 10.1212/WNL.54.10.1916. 10822429

[ref138] Hutton C , De Vita E, Ashburner J, Deichmann R, Turner R. Voxel-based cortical thickness measurements in MRI. NeuroImage. 2008:40(4):1701–1710. 10.1016/j.neuroimage.2008.01.027. 18325790 PMC2330066

[ref139] Hutton C , Draganski B, Ashburner J, Weiskopf N. A comparison between voxel-based cortical thickness and voxel-based morphometry in normal aging. NeuroImage. 2009:48(2):371–380. 10.1016/j.neuroimage.2009.06.043. 19559801 PMC2741580

[ref140] Irwin ZT , Awad MZ, Gonzalez CL, Nakhmani A, Bentley JN, Moore TA, Smithson KG, Guthrie BL, Walker HC. Latency of subthalamic nucleus deep brain stimulation-evoked cortical activity as a potential biomarker for postoperative motor side effects. Clin Neurophysiol. 2020:131(6):1221–1229. 10.1016/j.clinph.2020.02.021. 32299006 PMC7214089

[ref141] Jan C , Pessiglione M, Tremblay L, Tande D, Hirsch EC, Francois C. Quantitative analysis of dopaminergic loss in relation to functional territories in MPTP-treated monkeys. Eur J Neurosci. 2003:18(7):2082–2086. 10.1046/j.1460-9568.2003.02946.x. 14622241

[ref142] Jenkins IH , Fernandez W, Playford ED, Lees AJ, Frackowiak RSJ, Passingham RE, Brooks DJ. Impaired activation of the supplementary motor area in Parkinson's disease is reversed when akinesia is treated with apomorphine. Ann Neurol. 1992:32(6):749–757. 10.1002/ana.410320608. 1471865

[ref143] Johnson MD , Vitek JL, McIntyre CC. Pallidal stimulation that improves parkinsonian motor symptoms also modulates neuronal firing patterns in primary motor cortex in the MPTP-treated monkey. Exp Neurol. 2009:219(1):359–362. 10.1016/j.expneurol.2009.04.022. 19409895 PMC2730829

[ref144] Johnson LA , Xu W, Baker KB, Zhang J, Vitek JL. Modulation of motor cortex neuronal activity and motor behavior during subthalamic nucleus stimulation in the normal primate. J Neurophysiol. 2015:113(7):2549–2554. 10.1152/jn.00997.2014. 25673744 PMC4416594

[ref145] Johnson LA , Wang J, Nebeck SD, Zhang J, Johnson MD, Vitek JL. Direct activation of primary motor cortex during subthalamic but not pallidal deep brain stimulation. J Neurosci. 2020:40(10):2166–2177. 10.1523/JNEUROSCI.2480-19.2020. 32019827 PMC7055133

[ref146] Jung J , Lambon Ralph MA, Jackson RL. Subregions of DLPFC display graded yet distinct structural and functional connectivity. J Neurosci. 2022:42(15):3241–3252. 10.1523/JNEUROSCI.1216-21.2022. 35232759 PMC8994544

[ref147] Kacar A , Filipovic SR, Kresojevic N, Milanovic SD, Ljubisavljevic M, Kostic VS, Rothwell JC. History of exposure to dopaminergic medication does not affect motor cortex plasticity and excitability in Parkinson's disease. Clin Neurophysiol. 2013:124(4):697–707. 10.1016/j.clinph.2012.09.016. 23085389

[ref148] Kahan J , Urner M, Moran R, Flandin G, Marreiros A, Mancini L, White M, Thornton J, Yousry T, Zrinzo L, et al. Resting state functional MRI in Parkinson's disease: the impact of deep brain stimulation on 'effective' connectivity. Brain. 2014:137(4):1130–1144. 10.1093/brain/awu027. 24566670 PMC3959559

[ref149] Kanazawa M , Ohba H, Nishiyama S, Kakiuchi T, Tsukada H. Effect of MPTP on serotonergic neuronal systems and mitochondrial complex I activity in the living brain: a PET study on conscious rhesus monkeys. J Nucl Med. 2017:58(7):1111–1116. 10.2967/jnumed.116.189159. 28280215

[ref150] Kanno M , Matsumoto M, Togashi H, Yoshioka M, Mano Y. Effects of acute repetitive transcranial magnetic stimulation on dopamine release in the rat dorsolateral striatum. J Neurol Sci. 2004:217(1):73–81. 10.1016/j.jns.2003.08.013. 14675613

[ref151] Karimi F , Niu J, Gouweleeuw K, Almeida Q, Jiang N. Movement related EEG signatures associated with freezing of gait in Parkinson's disease: an integrative analysis. Brain Commun. 2021:3(4):fcab277. 10.1093/braincomms/fcab277. 34877535 PMC8643573

[ref152] Kerr CC , Van Albada SJ, Neymotin SA, Chadderdon GL, Robinson PA, Lytton WW. Cortical information flow in Parkinson's disease: a composite network/field model. Front Comput Neurosci. 2013:7:39. 10.3389/fncom.2013.00039. 23630492 PMC3635017

[ref153] Khanna P , Carmena JM. Beta band oscillations in motor cortex reflect neural population signals that delay movement onset. elife. 2017:6:e24573. 10.7554/eLife.24573. 28467303 PMC5468088

[ref154] Kish SJ . Biochemistry of Parkinson's disease: is a brain serotonergic deficiency a characteristic of idiopathic Parkinson's disease? Adv Neurol. 2003:91:39–49. 12442662

[ref155] Kishore A , Joseph T, Velayudhan B, Popa T, Meunier S. Early, severe and bilateral loss of LTP and LTD-like plasticity in motor cortex (M1) in de novo Parkinson's disease. Clin Neurophysiol. 2012:123(4):822–828. 10.1016/j.clinph.2011.06.034. 21945457

[ref156] Kitada T , Ardah MT, Haque ME. History of Parkinson's disease-associated gene, parkin: research over a quarter century in quest of finding the physiological substrate. Int J Mol Sci. 2023:24(23):16734. 10.3390/ijms242316734. PMC10706564 38069057

[ref157] Koshimori Y , Segura B, Christopher L, Lobaugh N, Duff-Canning S, Mizrahi R, Hamani C, Lang AE, Aminian K, Houle S, et al. Imaging changes associated with cognitive abnormalities in Parkinson’s disease. Brain Struct Funct. 2015:220(4):2249–2261. 10.1007/s00429-014-0785-x. 24816399 PMC4485490

[ref158] Kühn AA , Kupsch A, Schneider GH, Brown P. Reduction in subthalamic 8–35 Hz oscillatory activity correlates with clinical improvement in Parkinson's disease. Eur J Neurosci. 2006:23(7):1956–1960. 10.1111/j.1460-9568.2006.04717.x. 16623853

[ref159] Kurani AS , Seidler RD, Burciu RG, Comella CL, Corcos DM, Okun MS, MacKinnon CD, Vaillancourt DE. Subthalamic nucleus—sensorimotor cortex functional connectivity in de novo and moderate Parkinson's disease. Neurobiol Aging. 2015:36(1):462–469. 10.1016/j.neurobiolaging.2014.07.004. 25095723 PMC4268125

[ref160] Kuriakose R , Saha U, Castillo G, Udupa K, Ni Z, Gunraj C, Mazzella F, Hamani C, Lang AE, Moro E, et al. The nature and time course of cortical activation following subthalamic stimulation in Parkinson's disease. Cereb Cortex. 2010:20(8):1926–1936. 10.1093/cercor/bhp269. 20019146

[ref161] Lanoue AC , Dumitriu A, Myers RH, Soghomonian JJ. Decreased glutamic acid decarboxylase mRNA expression in prefrontal cortex in Parkinson's disease. Exp Neurol. 2010:226(1):207–217. 10.1016/j.expneurol.2010.09.001. 20832408 PMC3108022

[ref162] Lanoue AC , Blatt GJ, Soghomonian JJ. Decreased parvalbumin mRNA expression in dorsolateral prefrontal cortex in Parkinson's disease. Brain Res. 2013:1531:37–47. 10.1016/j.brainres.2013.07.025. 23891794 PMC3816277

[ref163] Lauro PM , Lee S, Akbar U, Asaad WF. Subthalamic-cortical network reorganization during Parkinson's tremor. J Neurosci. 2021:41:9844–9858. 34702744 10.1523/JNEUROSCI.0854-21.2021 PMC8612636

[ref164] Lauro PM , Lee S, Amaya DE, Liu DD, Akbar U, Asaad WF. Concurrent decoding of distinct neurophysiological fingerprints of tremor and bradykinesia in Parkinson's disease. elife. 2023:12:e84135. 10.7554/eLife.84135. PMC10264071 37249217

[ref165] Leemburg S , Canonica T, Luft A. Motor skill learning and reward consumption differentially affect VTA activation. Sci Rep. 2018:8(1):687. 10.1038/s41598-017-18716-w. 29330488 PMC5766527

[ref166] Lefaucheur JP . Motor cortex dysfunction revealed by cortical excitability studies in Parkinson's disease: influence of antiparkinsonian treatment and cortical stimulation. Clin Neurophysiol. 2005:116(2):244–253. 10.1016/j.clinph.2004.11.017. 15661100

[ref167] Lewis DA , Gonzalez-Burgos G. Pathophysiologically based treatment interventions in schizophrenia. Nat Med. 2006:12(9):1016–1022. 10.1038/nm1478. 16960576

[ref168] Lewis DA , Campbell MJ, Foote SL, Goldstein M, Morrison JH. The distribution of tyrosine hydroxylase-immunoreactive fibers in primate neocortex is widespread but regionally specific. J Neurosci. 1987:7(1):279–290. 10.1523/JNEUROSCI.07-01-00279.1987. 2879896 PMC6568855

[ref169] Li S , Arbuthnott GW, Jutras MJ, Goldberg JA, Jaeger D. Resonant antidromic cortical circuit activation as a consequence of high-frequency subthalamic deep-brain stimulation. J Neurophysiol. 2007:98(6):3525–3537. 10.1152/jn.00808.2007. 17928554

[ref170] Li Q , Ke Y, Chan Danny CW, Qian Z-M, Yung Ken KL, Ko H, Arbuthnott Gordon W, Yung W-H. Therapeutic deep brain stimulation in parkinsonian rats directly influences motor cortex. Neuron. 2012:76(5):1030–1041. 10.1016/j.neuron.2012.09.032. 23217750

[ref171] Li D , Wang E, Jia Y, Xu J, Zhang Z, Jiang Z, Luo W. Cortical complexity and gyrification patterns in Parkinson’s disease. Neuroreport. 2020:31(7):565–570. 10.1097/WNR.0000000000001448. 32251101

[ref172] Lidow MS . D1- and D2 dopaminergic receptors in the developing cerebral cortex of macaque monkey: a film autoradiographic study. Neuroscience. 1995:65(2):439–452. 10.1016/0306-4522(94)00475-K. 7777159

[ref173] Lidow MS , Goldman-Rakic PS, Gallager DW, Rakic P. Distribution of dopaminergic receptors in the primate cerebral cortex: quantitative autoradiographic analysis using [3H]raclopride, [3H]spiperone and [3H]SCH23390. Neuroscience. 1991:40(3):657–671. 10.1016/0306-4522(91)90003-7. 2062437

[ref174] Limousin P , Greene J, Pollak P, Rothwell J, Benabid AL, Frackowiak R. Changes in cerebral activity pattern due to subthalamic nucleus or internal pallidum stimulation in Parkinson's disease. Ann Neurol. 1997:42(3):283–291. 10.1002/ana.410420303. 9307248

[ref175] Litvak V , Jha A, Eusebio A, Oostenveld R, Foltynie T, Limousin P, Zrinzo L, Hariz MI, Friston K, Brown P. Resting oscillatory cortico-subthalamic connectivity in patients with Parkinson's disease. Brain. 2011:134(2):359–374. 10.1093/brain/awq332. 21147836

[ref176] Liu AK , Chang RC, Pearce RK, Gentleman SM. Nucleus basalis of Meynert revisited: anatomy, history and differential involvement in Alzheimer's and Parkinson's disease. Acta Neuropathol. 2015:129(4):527–540. 10.1007/s00401-015-1392-5. 25633602 PMC4366544

[ref177] Louie KH , Gilron R, Yaroshinsky MS, Morrison MA, Choi J, de Hemptinne C, Little S, Starr PA, Wang DD. Cortico-subthalamic field potentials support classification of the natural gait cycle in Parkinson's disease and reveal individualized spectral signatures. eNeuro. 2022:ENEURO.0325-22.2022. 10.1523/ENEURO.0325-22.2022. PMC9663205 36270803

[ref178] Luft AR , Schwarz S. Dopaminergic signals in primary motor cortex. Int J Dev Neurosci. 2009:27(5):415–421. 10.1016/j.ijdevneu.2009.05.004. 19446627

[ref179] Luo C , Song W, Chen Q, Zheng Z, Chen K, Cao B, Yang J, Li J, Huang X, Gong Q, et al. Reduced functional connectivity in early-stage drug-naive Parkinson's disease: a resting-state fMRI study. Neurobiol Aging. 2014:35(2):431–441. 10.1016/j.neurobiolaging.2013.08.018. 24074808

[ref180] Luoma J , Pekkonen E, Airaksinen K, Helle L, Nurminen J, Taulu S, Makela JP. Spontaneous sensorimotor cortical activity is suppressed by deep brain stimulation in patients with advanced Parkinson's disease. Neurosci Lett. 2018:683:48–53. 10.1016/j.neulet.2018.06.041. 29940326

[ref181] Lytton WW , Arle J, Bobashev G, Ji S, Klassen TL, Marmarelis VZ, Schwaber J, Sherif MA, Sanger TD. Multiscale modeling in the clinic: diseases of the brain and nervous system. Brain Inform. 2017:4(4):219–230. 10.1007/s40708-017-0067-5. 28488252 PMC5709279

[ref182] MacKinnon CD , Gilley EA, Weis-McNulty A, Simuni T. Pathways mediating abnormal intracortical inhibition in Parkinson's disease. Ann Neurol. 2005:58(4):516–524. 10.1002/ana.20599. 16178015

[ref183] Madelein van der Stouwe AM , Nieuwhof F, Helmich RC. Tremor pathophysiology: lessons from neuroimaging. Curr Opin Neurol. 2020:33(4):474–481. 10.1097/WCO.0000000000000829. 32657888

[ref184] Magnani G , Cursi M, Leocani L, Volonte MA, Comi G. Acute effects of L-dopa on event-related desynchronization in Parkinson's disease. Neurol Sci. 2002:23(3):91–97. 10.1007/s100720200033. 12391492

[ref185] Magno LAV , Tenza-Ferrer H, Collodetti M, Aguiar MFG, Rodrigues APC, Silva RS, Silva JP, Nicolau NF, Rosa DVF, Birbrair A, et al. Optogenetic stimulation of the M2 cortex reverts motor dysfunction in a mouse model of Parkinson's disease. J Neurosci. 2019:39(17):3234–3248. 10.1523/JNEUROSCI.2277-18.2019. 30782975 PMC6788829

[ref186] Maillet A , Krack P, Lhommee E, Metereau E, Klinger H, Favre E, Le Bars D, Schmitt E, Bichon A, Pelissier P, et al. The prominent role of serotonergic degeneration in apathy, anxiety and depression in de novo Parkinson's disease. Brain. 2016:139(9):2486–2502. 10.1093/brain/aww162. 27538418

[ref187] Mallet N , Pogosyan A, Marton LF, Bolam JP, Brown P, Magill PJ. Parkinsonian beta oscillations in the external globus pallidus and their relationship with subthalamic nucleus activity. J Neurosci. 2008:28(52):14245–14258. 10.1523/JNEUROSCI.4199-08.2008. 19109506 PMC4243385

[ref188] Mally J , Stone TW. Improvement in parkinsonian symptoms after repetitive transcranial magnetic stimulation. J Neurol Sci. 1999:162(2):179–184. 10.1016/S0022-510X(98)00318-9. 10202984

[ref189] Mandir AS , Watts RL. Changes in primary motor cortex neuronal activity associated with increased reaction time and movement time in MPTP parkinsonism. Mov Disord. 1990:5(Suppl. 1):77.

[ref190] Masilamoni GJ , Weinkle A, Papa SM, Smith Y. Cortical serotonergic and Catecholaminergic denervation in MPTP-treated parkinsonian monkeys. Cereb Cortex. 2022:32(9):1804–1822. 10.1093/cercor/bhab313. 34519330 PMC9070356

[ref191] Mathai A , Ma Y, Paré J-F, Villalba RM, Wichmann T, Smith Y. Reduced cortical innervation of the subthalamic nucleus in MPTP-treated parkinsonian monkeys. Brain. 2015:138(4):946–962. 10.1093/brain/awv018. 25681412 PMC5014077

[ref192] Matthews DC , Lerman H, Lukic A, Andrews RD, Mirelman A, Wernick MN, Giladi N, Strother SC, Evans KC, Cedarbaum JM, et al. FDG PET Parkinson’s disease-related pattern as a biomarker for clinical trials in early stage disease. NeuroImage Clin. 2018:20:572–579. 10.1016/j.nicl.2018.08.006. 30186761 PMC6120603

[ref193] McCairn KW , Turner RS. Pallidal stimulation suppresses pathological dysrhythmia in the parkinsonian motor cortex. J Neurophysiol. 2015:113(7):2537–2548. 10.1152/jn.00701.2014. 25652922 PMC4416560

[ref194] McFarland NR , Haber SN. Thalamic relay nuclei of the basal ganglia form both reciprocal and nonreciprocal cortical connections, linking multiple frontal cortical areas. J Neurosci. 2002:22(18):8117–8132. 10.1523/JNEUROSCI.22-18-08117.2002. 12223566 PMC6758100

[ref195] Meier JM , Perdikis D, Blickensdorfer A, Stefanovski L, Liu Q, Maith O, Dinkelbach HU, Baladron J, Hamker FH, Ritter P. Virtual deep brain stimulation: multiscale co-simulation of a spiking basal ganglia model and a whole-brain mean-field model with the virtual brain. Exp Neurol. 2022:354:114111. 10.1016/j.expneurol.2022.114111. 35569510

[ref196] Meissner SN , Krause V, Sudmeyer M, Hartmann CJ, Pollok B. The significance of brain oscillations in motor sequence learning: insights from Parkinson's disease. Neuroimage Clin. 2018:20:448–457. 10.1016/j.nicl.2018.08.009. 30128283 PMC6095950

[ref197] Mertiens S , Sure M, Schnitzler A, Florin E. Alterations of PAC-based resting state networks in Parkinson's disease are partially alleviated by levodopa medication. Front Syst Neurosci. 2023:17:1219334. 10.3389/fnsys.2023.1219334. 37588811 PMC10427244

[ref198] Milardi D , Gaeta M, Marino S, Arrigo A, Vaccarino G, Mormina E, Rizzo G, Milazzo C, Finocchio G, Baglieri A, et al. Basal ganglia network by constrained spherical deconvolution: a possible cortico-pallidal pathway? Mov Disord. 2015:30(3):342–349. 10.1002/mds.25995. 25156805

[ref199] Miller AM , Miocinovic S, Swann NC, Rajagopalan SS, Darevsky DM, Re G, Hemptinne C, Ostrem JL, Starr PA. Effect of levodopa on electroencephalographic biomarkers of the parkinsonian state. J Neurophysiol. 2019:122(1):290–299. 10.1152/jn.00141.2019. 31066605 PMC6689788

[ref200] Miocinovic S , de Hemptinne C, Chen W, Isbaine F, Willie JT, Ostrem JL, Starr PA. Cortical potentials evoked by subthalamic stimulation demonstrate a short latency Hyperdirect pathway in humans. J Neurosci. 2018:38(43):9129–9141. 10.1523/JNEUROSCI.1327-18.2018. 30201770 PMC6199405

[ref201] Mitchell T , Lehéricy S, Chiu SY, Strafella AP, Stoessl AJ, Vaillancourt DE. Emerging neuroimaging biomarkers across disease stage in Parkinson disease: a review. JAMA Neurol. 2021:78(10):1262–1272. 10.1001/jamaneurol.2021.1312. 34459865 PMC9017381

[ref202] Molina-Luna K , Pekanovic A, Rohrich S, Hertler B, Schubring-Giese M, Rioult-Pedotti MS, Luft AR. Dopamine in motor cortex is necessary for skill learning and synaptic plasticity. PLoS One. 2009:4(9):e7082. 10.1371/journal.pone.0007082. 19759902 PMC2738964

[ref203] Moriguchi S , Kimura Y, Ichise M, Arakawa R, Takano H, Seki C, Ikoma Y, Takahata K, Nagashima T, Yamada M, et al. PET quantification of the norepinephrine transporter in human brain with (S,S)-(18)F-FMeNER-D(2). J Nucl Med. 2017:58(7):1140–1145. 10.2967/jnumed.116.178913. 27980046

[ref204] Moro E , Schwalb JM, Piboolnurak P, Poon YY, Hamani C, Hung SW, Arenovich T, Lang AE, Chen R, Lozano AM. Unilateral subdural motor cortex stimulation improves essential tremor but not Parkinson's disease. Brain. 2011:134(7):2096–2105. 10.1093/brain/awr072. 21646329

[ref205] Mure H , Hirano S, Tang CC, Isaias IU, Antonini A, Ma Y, Dhawan V, Eidelberg D. Parkinson's disease tremor-related metabolic network: characterization, progression, and treatment effects. NeuroImage. 2011:54(2):1244–1253. 10.1016/j.neuroimage.2010.09.028. 20851193 PMC2997135

[ref206] Muthuraman M , Bange M, Koirala N, Ciolac D, Pintea B, Glaser M, Tinkhauser G, Brown P, Deuschl G, Groppa S. Cross-frequency coupling between gamma oscillations and deep brain stimulation frequency in Parkinson's disease. Brain. 2020:143(11):3393–3407. 10.1093/brain/awaa297. 33150359 PMC7116448

[ref207] Neely KA , Kurani AS, Shukla P, Planetta PJ, Wagle Shukla A, Goldman JG, Corcos DM, Okun MS, Vaillancourt DE. Functional brain activity relates to 0–3 and 3–8 Hz force oscillations in essential tremor. Cereb Cortex. 2015:25(11):4191–4202. 10.1093/cercor/bhu142. 24962992 PMC4816778

[ref208] Ni Z , Bahl N, Gunraj CA, Mazzella F, Chen R. Increased motor cortical facilitation and decreased inhibition in Parkinson disease. Neurology. 2013:80(19):1746–1753. 10.1212/WNL.0b013e3182919029. 23576626 PMC3719423

[ref209] Nishijima H , Ueno T, Funamizu Y, Ueno S, Tomiyama M. Levodopa treatment and dendritic spine pathology. Mov Disord. 2018:33(6):877–888. 10.1002/mds.27172. 28880414 PMC6667906

[ref211] Ogawa T , Matson WR, Beal MF, Myers RH, Bird ED, Milbury P, Saso S. Kynurenine pathway abnormalities in Parkinson's disease. Neurology. 1992:42(9):1702–1706. 10.1212/WNL.42.9.1702. 1513457

[ref212] O'Keeffe AB , Malekmohammadi M, Sparks H, Pouratian N. Synchrony drives motor cortex Beta bursting, waveform dynamics, and phase-amplitude coupling in Parkinson's disease. J Neurosci. 2020:40(30):5833–5846. 10.1523/JNEUROSCI.1996-19.2020. 32576623 PMC7380967

[ref213] Okkels N , Horsager J, Labrador-Espinosa M, Kjeldsen PL, Damholdt MF, Mortensen J, Vestergard K, Knudsen K, Andersen KB, Fedorova TD, et al. Severe cholinergic terminal loss in newly diagnosed dementia with Lewy bodies. Brain. 2023:146(9):3690–3704. 10.1093/brain/awad192. 37279796

[ref214] Oswal A , Cao C, Yeh CH, Neumann WJ, Gratwicke J, Akram H, Horn A, Li D, Zhan S, Zhang C, et al. Neural signatures of hyperdirect pathway activity in Parkinson's disease. Nat Commun. 2021:12(1):5185. 10.1038/s41467-021-25366-0. 34465771 PMC8408177

[ref215] Ozkurt TE , Akram H, Zrinzo L, Limousin P, Foltynie T, Oswal A, Litvak V. Identification of nonlinear features in cortical and subcortical signals of Parkinson's disease patients via a novel efficient measure. NeuroImage. 2020:223:117356. 10.1016/j.neuroimage.2020.117356. 32916287 PMC8417768

[ref216] Parent M , Parent A. Single-axon tracing and three-dimensional reconstruction of Centre median-parafascicular thalamic neurons in primates. J Comp Neurol. 2005:481(1):127–144. 10.1002/cne.20348. 15558721

[ref217] Parker PRL , Lalive AL, Kreitzer AC. Pathway-specific remodeling of thalamostriatal synapses in parkinsonian mice. Neuron. 2016:89(4):734–740. 10.1016/j.neuron.2015.12.038. 26833136 PMC4760843

[ref218] Parr-Brownlie LC , Hyland BI. Bradykinesia induced by dopamine D2 receptor blockade is associated with reduced motor cortex activity in the rat. J Neurosci. 2005:25(24):5700–5709. 10.1523/JNEUROSCI.0523-05.2005. 15958736 PMC6724886

[ref219] Parr-Brownlie LC , Itoga CA, Walters JR, Underwood CF. Oscillatory waveform sharpness asymmetry changes in motor thalamus and motor cortex in a rat model of Parkinson's disease. Exp Neurol. 2022:354:114089. 10.1016/j.expneurol.2022.114089. 35461830 PMC11345867

[ref220] Pascual-Leone A , Valls-Sole J, Brasil-Neto JP, Cammarota A, Grafman J, Hallett M. Akinesia in Parkinson's disease. II. Effects of subthreshold repetitive transcranial motor cortex stimulation. Neurology. 1994:44(5):892–898. 10.1212/WNL.44.5.892. 8190293

[ref221] Pasquereau B , Turner RS. Primary motor cortex of the parkinsonian monkey: differential effects on the spontaneous activity of pyramidal tract-type neurons. Cereb Cortex. 2011:21(6):1362–1378. 10.1093/cercor/bhq217. 21045003 PMC3097989

[ref222] Pasquereau B , Turner RS. Primary motor cortex of the parkinsonian monkey: altered neuronal responses to muscle stretch. Front Syst Neurosci. 2013:7:98. 10.3389/fnsys.2013.00098. 24324412 PMC3840326

[ref223] Pasquereau B , DeLong MR, Turner RS. Primary motor cortex of the parkinsonian monkey: altered encoding of active movement. Brain. 2016:139(1):127–143. 10.1093/brain/awv312. 26490335 PMC4794619

[ref224] Pasquini J , Brooks DJ, Pavese N. The cholinergic brain in Parkinson's disease. Mov Disord Clin Pract. 2021:8(7):1012–1026. 10.1002/mdc3.13319. 34631936 PMC8485627

[ref225] Pauls KAM , Korsun O, Nenonen J, Nurminen J, Liljestrom M, Kujala J, Pekkonen E, Renvall H. Cortical beta burst dynamics are altered in Parkinson's disease but normalized by deep brain stimulation. NeuroImage. 2022:257:119308. 10.1016/j.neuroimage.2022.119308. 35569783

[ref226] Pedersen KM , Marner L, Pakkenberg H, Pakkenberg B. No global loss of neocortical neurons in Parkinson's disease: a quantitative stereological study. Mov Disord. 2005:20(2):164–171. 10.1002/mds.20289. 15468109

[ref227] Peng GCY , Alber M, Tepole AB, Cannon WR, De S, Dura-Bernal S, Garikipati K, Karniadakis G, Lytton WW, Perdikaris P, et al. Multiscale modeling meets machine learning: what can we learn? Arch Comput Methods Eng. 2021:28(3):1017–1037. 10.1007/s11831-020-09405-5. 34093005 PMC8172124

[ref228] Perez-Otano I , Oset C, Luquin MR, Herrero MT, Obeso JA, Del Rio J. MPTP-induced parkinsonism in primates: pattern of striatal dopamine loss following acute and chronic administration. Neurosci Lett. 1994:175(1–2):121–125. 10.1016/0304-3940(94)91094-4. 7970192

[ref229] Pfurtscheller G , Pichler-Zalaudek K, Ortmayr B, Diez J, Reisecker F. Postmovement beta synchronization in patients with Parkinson's disease. J Clin Neurophysiol. 1998:15(3):243–250. 10.1097/00004691-199805000-00008. 9681562

[ref230] Piano C , Bove F, Mulas D, Di Stasio E, Fasano A, Bentivoglio AR, Daniele A, Cioni B, Calabresi P, Tufo T. Extradural motor cortex stimulation in Parkinson's disease: long-term clinical outcome. Brain Sci. 2021:11(4). 10.3390/brainsci11040416. PMC8067040 33810277

[ref231] Pifl C , Schingnitz G, Hornykiewicz O. Effect of 1-methyl-4-phenyl-1,2,3,6-tetrahydropyridine on the regional distribution of brain monoamines in the rhesus monkey. Neuroscience. 1991:44(3):591–605. 10.1016/0306-4522(91)90080-8. 1754053

[ref232] Planetta PJ , Kurani AS, Shukla P, Prodoehl J, Corcos DM, Comella CL, McFarland NR, Okun MS, Vaillancourt DE. Distinct functional and macrostructural brain changes in Parkinson's disease and multiple system atrophy. Hum Brain Mapp. 2015:36(3):1165–1179. 10.1002/hbm.22694. 25413603 PMC4950674

[ref233] Plateau V , Baufreton J, Le Bon-Jego M. Age-dependent modulation of layer V pyramidal neuron excitability in the mouse primary motor cortex by D1 receptor agonists and antagonists. Neuroscience. 2023:536:21–35. 10.1016/j.neuroscience.2023.11.006. 37952579

[ref234] Playford ED , Jenkins IH, Passingham RE, Nutt J, Frackowiak RSJ, Brooks DJ. Impaired mesial frontal and putamen activation in Parkinson's disease: a positron emission tomography study. Ann Neurol. 1992:32(2):151–161. 10.1002/ana.410320206. 1510355

[ref235] Politis M , Wu K, Loane C, Kiferle L, Molloy S, Brooks DJ, Piccini P. Staging of serotonergic dysfunction in Parkinson's disease: an in vivo 11C-DASB PET study. Neurobiol Dis. 2010:40(1):216–221. 10.1016/j.nbd.2010.05.028. 20594979

[ref236] Politis M , Wu K, Loane C, Quinn NP, Brooks DJ, Oertel WH, Bjorklund A, Lindvall O, Piccini P. Serotonin neuron loss and nonmotor symptoms continue in Parkinson's patients treated with dopamine grafts. Sci Transl Med. 2012:4(128):128ra141. 10.1126/scitranslmed.3003391. 22491951

[ref237] Pollok B , Krause V, Martsch W, Wach C, Schnitzler A, Sudmeyer M. Motor-cortical oscillations in early stages of Parkinson's disease. J Physiol. 2012:590(13):3203–3212. 10.1113/jphysiol.2012.231316. 22547636 PMC3406400

[ref238] Pollok B , Kamp D, Butz M, Wojtecki L, Timmermann L, Sudmeyer M, Krause V, Schnitzler A. Increased SMA-M1 coherence in Parkinson's disease - pathophysiology or compensation? Exp Neurol. 2013:247:178–181. 10.1016/j.expneurol.2013.04.013. 23664959

[ref239] Prodoehl J , Planetta PJ, Kurani AS, Comella CL, Corcos DM, Vaillancourt DE. Differences in brain activation between tremor- and nontremor-dominant Parkinson disease. JAMA Neurol. 2013:70(1):100–106. 10.1001/jamaneurol.2013.582. 23318516 PMC3645004

[ref240] Prokic EJ , Stanford IM, Woodhall GL, Williams AC, Hall SD. Bradykinesia is driven by cumulative Beta power during continuous movement and alleviated by Gabaergic modulation in Parkinson's disease. Front Neurol. 2019:10:1298. 10.3389/fneur.2019.01298. 31920922 PMC6933612

[ref241] Pyatigorskaya N , Gallea C, Garcia-Lorenzo D, Vidailhet M, Lehericy S. A review of the use of magnetic resonance imaging in Parkinson's disease. Ther Adv Neurol Disord. 2014:7(4):206–220. 10.1177/1756285613511507. 25002908 PMC4082302

[ref242] Qasim SE , de Hemptinne C, Swann NC, Miocinovic S, Ostrem JL, Starr PA. Electrocorticography reveals beta desynchronization in the basal ganglia-cortical loop during rest tremor in Parkinson's disease. Neurobiol Dis. 2016:86:177–186. 10.1016/j.nbd.2015.11.023. 26639855 PMC4842026

[ref243] Ray Chaudhuri K , Leta V, Bannister K, Brooks DJ, Svenningsson P. The noradrenergic subtype of Parkinson disease: from animal models to clinical practice. Nat Rev Neurol. 2023:19(6):333–345. 10.1038/s41582-023-00802-5. 37142796

[ref244] Richfield EK , Young AB, Penney JB. Comparative distributions of dopamine D-1 and D-2 receptors in the cerebral cortex of rats, cats, and monkeys. J Comp Neurol. 1989:286(4):409–426. 10.1002/cne.902860402. 2506254

[ref245] Ridding MC , Inzelberg R, Rothwell JC. Changes in excitability of motor cortical circuitry in patients with Parkinson's disease. Ann Neurol. 1995:37(2):181–188. 10.1002/ana.410370208. 7847860

[ref246] Rioult-Pedotti MS , Pekanovic A, Atiemo CO, Marshall J, Luft AR. Dopamine promotes motor cortex plasticity and motor skill learning via PLC activation. PLoS One. 2015:10(5):e0124986. 10.1371/journal.pone.0124986. 25938462 PMC4418826

[ref247] Rothwell JC , Edwards MJ. Parkinson's disease. Handb Clin Neurol. 2013:116:535–542. 10.1016/B978-0-444-53497-2.00042-5. 24112921

[ref248] Sabatini U , Boulanouar K, Fabre N, Martin F, Carel C, Colonnese C, Bozzao L, Berry I, Montastruc J, Chollet F, et al. Cortical motor reorganization in akinetic patients with Parkinson's disease: a functional MRI study. Brain. 2000:123(2):394–403. 10.1093/brain/123.2.394. 10648446

[ref249] Sanders TH , Jaeger D. Optogenetic stimulation of cortico-subthalamic projections is sufficient to ameliorate bradykinesia in 6-ohda lesioned mice. Neurobiol Dis. 2016:95:225–237. 10.1016/j.nbd.2016.07.021. 27452483 PMC5010926

[ref250] Sarmiento ACP , García YB, Gómez JFO. Active information storage in Parkinson’s disease: a resting state fMRI study over the sensorimotor cortex. Brain Imaging Behav. 2020:14(4):1143–1153. 10.1007/s11682-019-00037-3. 30684153

[ref251] Scatton B , Javoy-Agid F, Rouquier L, Dubois B, Agid Y. Reduction of cortical dopamine, noradrenaline, serotonin and their metabolites in Parkinson's disease. Brain Res. 1983:275(2):321–328. 10.1016/0006-8993(83)90993-9. 6626985

[ref252] Schneider JS , Kovelowski CJ 2nd. Chronic exposure to low doses of MPTP. I. Cognitive deficits in motor asymptomatic monkeys. Brain Res. 1990:519(1–2):122–128. 10.1016/0006-8993(90)90069-N. 2397401

[ref253] Shah C , Beall EB, Frankemolle AM, Penko A, Phillips MD, Lowe MJ, Alberts JL. Exercise therapy for Parkinson's disease: pedaling rate is related to changes in motor connectivity. Brain Connect. 2016:6(1):25–36. 10.1089/brain.2014.0328. 26414696 PMC4744893

[ref254] Sharma A , Vidaurre D, Vesper J, Schnitzler A, Florin E. Differential dopaminergic modulation of spontaneous cortico-subthalamic activity in Parkinson's disease. elife. 2021:10:e66057. 10.7554/eLife.66057. PMC8177893 34085932

[ref255] Shepherd GMG , Yamawaki N. Untangling the cortico-thalamo-cortical loop: cellular pieces of a knotty circuit puzzle. Nat Rev Neurosci. 2021:22(7):389–406. 10.1038/s41583-021-00459-3. 33958775 PMC9006917

[ref256] Shimamoto H , Takasaki K, Shigemori M, Imaizumi T, Ayabe M, Shoji H. Therapeutic effect and mechanism of repetitive transcranial magnetic stimulation in Parkinson's disease. J Neurol. 2001:248(S3):III48–III52. 10.1007/PL00007826. 11697688

[ref257] Shimamoto SA , Ryapolova-Webb ES, Ostrem JL, Galifianakis NB, Miller KJ, Starr PA. Subthalamic nucleus neurons are synchronized to primary motor cortex local field potentials in Parkinson's disease. J Neurosci. 2013:33(17):7220–7233. 10.1523/JNEUROSCI.4676-12.2013. 23616531 PMC3673303

[ref258] Siebner HR , Mentschel C, Auer C, Conrad B. Repetitive transcranial magnetic stimulation has a beneficial effect on bradykinesia in Parkinson's disease. Neuroreport. 1999:10(3):589–594. 10.1097/00001756-199902250-00027. 10208595

[ref259] Silberstein P , Pogosyan A, Kuhn AA, Hotton G, Tisch S, Kupsch A, Dowsey-Limousin P, Hariz MI, Brown P. Cortico-cortical coupling in Parkinson's disease and its modulation by therapy. Brain. 2005:128(6):1277–1291. 10.1093/brain/awh480. 15774503

[ref260] Smith Y , Wichmann T. The cortico-pallidal projection: an additional route for cortical regulation of the basal ganglia circuitry. Mov Disord. 2015:30(3):293–295. 10.1002/mds.26095. 25476969 PMC4357539

[ref261] Smith Y , Galvan A, Ellender TJ, Doig N, Villalba RM, Huerta-Ocampo I, Wichmann T, Bolam JP. The thalamostriatal system in normal and diseased states. Front Syst Neurosci. 2014:8:5. 10.3389/fnsys.2014.00005. 24523677 PMC3906602

[ref262] Solis O , Limon DI, Flores-Hernandez J, Flores G. Alterations in dendritic morphology of the prefrontal cortical and striatum neurons in the unilateral 6-OHDA-rat model of Parkinson's disease. Synapse. 2007:61(6):450–458. 10.1002/syn.20381. 17372982

[ref263] Sommerauer M , Fedorova TD, Hansen AK, Knudsen K, Otto M, Jeppesen J, Frederiksen Y, Blicher JU, Geday J, Nahimi A, et al. Evaluation of the noradrenergic system in Parkinson's disease: an 11C-MeNER PET and neuromelanin MRI study. Brain. 2018:141(2):496–504. 10.1093/brain/awx348. 29272343

[ref264] Spraker MB , Prodoehl J, Corcos DM, Comella CL, Vaillancourt DE. Basal ganglia hypoactivity during grip force in drug naive Parkinson's disease. Hum Brain Mapp. 2010:31(12):1928–1941. 10.1002/hbm.20987. 20225221 PMC6870615

[ref265] Steidel K , Ruppert MC, Greuel A, Tahmasian M, Maier F, Hammes J, Tv E, Timmermann L, Tittgemeyer M, Drzezga A, et al. Longitudinal trimodal imaging of midbrain-associated network degeneration in Parkinson’s disease. npj Park Dis. 2022:8(1):79. 10.1038/s41531-022-00341-8. PMC9218128 35732679

[ref266] Sterling NW , Wang M, Zhang L, Lee E-Y, Du G, Lewis MM, Styner M, Huang X. Stage-dependent loss of cortical gyrification as Parkinson disease “unfolds”. Neurology. 2016:86(12):1143–1151. 10.1212/WNL.0000000000002492. 26888982 PMC4820131

[ref267] Sterling NW , Du G, Lewis MM, Swavely S, Kong L, Styner M, Huang X. Cortical gray and subcortical white matter associations in Parkinson's disease. Neurobiol Aging. 2017:49:100–108. 10.1016/j.neurobiolaging.2016.09.015. 27776262 PMC5154847

[ref268] Stoffers D , Bosboom JL, Wolters E, Stam CJ, Berendse HW. Dopaminergic modulation of cortico-cortical functional connectivity in Parkinson's disease: an MEG study. Exp Neurol. 2008:213(1):191–195. 10.1016/j.expneurol.2008.05.021. 18590728

[ref269] Strafella AP , Ko JH, Grant J, Fraraccio M, Monchi O. Corticostriatal functional interactions in Parkinson's disease: a rTMS/[11C]raclopride PET study. Eur J Neurosci. 2005:22(11):2946–2952. 10.1111/j.1460-9568.2005.04476.x. 16324129 PMC2967526

[ref270] Strick PL , Dum RP, Rathelot J-A. The cortical motor areas and the emergence of motor skills: a neuroanatomical perspective. Annu Rev Neurosci. 2021:44(1):425–447. 10.1146/annurev-neuro-070918-050216. 33863253

[ref271] Sun S , Wang X, Shi X, Fang H, Sun Y, Li M, Han H, He Q, Wang X, Zhang X, et al. Neural pathway connectivity and discharge changes between M1 and STN in hemiparkinsonian rats. Brain Res Bull. 2023:196:1–19. 10.1016/j.brainresbull.2023.03.002. 36878325

[ref272] Swann NC , Hemptinne C, Aron AR, Ostrem JL, Knight RT, Starr PA. Elevated synchrony in Parkinson disease detected with electroencephalography. Ann Neurol. 2015:78(5):742–750. 10.1002/ana.24507. 26290353 PMC4623949

[ref273] Tanaka M , Yanagisawa T, Fukuma R, Tani N, Oshino S, Mihara M, Hattori N, Kajiyama Y, Hashimoto R, Ikeda M, et al. Magnetoencephalography detects phase-amplitude coupling in Parkinson's disease. Sci Rep. 2022:12(1):1835. 10.1038/s41598-022-05901-9. 35115607 PMC8813926

[ref274] Thobois S , Dominey P, Fraix V, Mertens P, Guenot M, Zimmer L, Pollak P, Benabid A-L, Broussolle E. Effects of subthalamic nucleus stimulation on actual and imagined movement in Parkinson's disease: a PET study. J Neurol. 2002:249(12):1689–1698. 10.1007/s00415-002-0906-y. 12529791

[ref275] Timmermann L , Florin E, Reck C. Pathological cerebral oscillatory activity in Parkinson's disease: a critical review on methods, data and hypotheses. Expert Rev Med Devices. 2007:4(5):651–661. 10.1586/17434440.4.5.651. 17850199

[ref276] Tosserams A , Weerdesteyn V, Bal T, Bloem BR, Solis-Escalante T, Nonnekes J. Cortical correlates of gait compensation strategies in Parkinson disease. Ann Neurol. 2022:91(3):329–341. 10.1002/ana.26306. 35067999 PMC9306676

[ref277] Turner RS , Grafton ST, McIntosh AR, DeLong MR, Hoffman JM. The functional anatomy of parkinsonian bradykinesia. NeuroImage. 2003:19(1):163–179. 10.1016/S1053-8119(03)00059-4. 12781736

[ref278] Uchihara T , Giasson BI. Propagation of alpha-synuclein pathology: hypotheses, discoveries, and yet unresolved questions from experimental and human brain studies. Acta Neuropathol. 2016:131(1):49–73. 10.1007/s00401-015-1485-1. 26446103 PMC4698305

[ref297] van Wijk BC , Beudel M, Jha A, Oswal A, Foltynie T, Hariz MI, Limousin P, Zrinzo L, Aziz TZ, Green AL, et al. Subthalamic nucleus phase-amplitude coupling correlates with motor impairment in Parkinson's disease. Clin Neurophysiol. 2016:127(4):2010–2019. 10.1016/j.clinph.2016.01.015. 26971483 PMC4803022

[ref210] van Nuland AJM , den Ouden HEM, Zach H, Dirkx MFM, van Asten JJA, Scheenen TWJ, Toni I, Cools R, Helmich RC. GABAergic changes in the thalamocortical circuit in Parkinson's disease. Hum Brain Mapp. 2020:41(4):1017–1029. 10.1002/hbm.24857. 31721369 PMC7267977

[ref279] Valverde S , Vandecasteele M, Piette C, Derousseaux W, Gangarossa G, Arbelaiz AA, Touboul J, Degos B, Venance L. Deep brain stimulation-guided optogenetic rescue of parkinsonian symptoms. Nat Commun. 2020:11(1):2388. 10.1038/s41467-020-16046-6. 32404907 PMC7220902

[ref280] Vardy AN , van Wegen EE, Kwakkel G, Berendse HW, Beek PJ, Daffertshofer A. Slowing of M1 activity in Parkinson’s disease during rest and movement – An MEG study. Clin Neurophysiol. 2011:122(4):789–795. 10.1016/j.clinph.2010.10.034. 21109487

[ref281] Villalba RM , Wichmann T, Smith Y. Neuronal loss in the caudal intralaminar thalamic nuclei in a primate model of Parkinson's disease. Brain Struct Funct. 2014:219(1):381–394. 10.1007/s00429-013-0507-9. 23508713 PMC3864539

[ref282] Villalba RM , Pare JF, Lee S, Lee S, Smith Y. Thalamic degeneration in MPTP-treated parkinsonian monkeys: impact upon glutamatergic innervation of striatal cholinergic interneurons. Brain Struct Funct. 2019:224(9):3321–3338. 10.1007/s00429-019-01967-w. 31679085 PMC6878768

[ref283] Villalba RM , Behnke JA, Pare JF, Smith Y. Comparative ultrastructural analysis of Thalamocortical innervation of the primary motor cortex and supplementary motor area in control and MPTP-treated parkinsonian monkeys. Cereb Cortex. 2021:31(7):3408–3425. 10.1093/cercor/bhab020. 33676368 PMC8599722

[ref284] Vinding MC , Tsitsi P, Piitulainen H, Waldthaler J, Jousmaki V, Ingvar M, Svenningsson P, Lundqvist D. Attenuated beta rebound to proprioceptive afferent feedback in Parkinson's disease. Sci Rep. 2019:9(1):2604. 10.1038/s41598-019-39204-3. 30796340 PMC6385616

[ref285] Vinding MC , Tsitsi P, Waldthaler J, Oostenveld R, Ingvar M, Svenningsson P, Lundqvist D. Reduction of spontaneous cortical beta bursts in Parkinson's disease is linked to symptom severity. Brain Commun. 2020:2(1):fcaa052. 10.1093/braincomms/fcaa052. 32954303 PMC7425382

[ref286] Vinding MC , Waldthaler J, Eriksson A, Manting CL, Ferreira D, Ingvar M, Svenningsson P, Lundqvist D. Oscillatory and non-oscillatory features of the magnetoencephalic sensorimotor rhythm in Parkinson's disease. NPJ Parkinsons Dis. 2024:10(1):51. 10.1038/s41531-024-00669-3. 38443402 PMC10915140

[ref287] Vucic S , Stanley Chen KH, Kiernan MC, Hallett M, Benninger DH, Di Lazzaro V, Rossini PM, Benussi A, Berardelli A, Curra A, et al. Clinical diagnostic utility of transcranial magnetic stimulation in neurological disorders. Updated report of an IFCN committee. Clin Neurophysiol. 2023:150:131–175. 10.1016/j.clinph.2023.03.010. 37068329 PMC10192339

[ref288] Wakabayashi K , Hansen LA, Masliah E. Cortical Lewy body-containing neurons are pyramidal cells: laser confocal imaging of double-immunolabeled sections with anti-ubiquitin and SMI32. Acta Neuropathol. 1995:89(5):404–408. 10.1007/BF00307643. 7618438

[ref289] Wang HC , Lees AJ, Brown P. Impairment of EEG desynchronisation before and during movement and its relation to bradykinesia in Parkinson's disease. J Neurol Neurosurg Psychiatry. 1999:66(4):442–446. 10.1136/jnnp.66.4.442. 10201414 PMC1736289

[ref290] Wang Z , Yan J, Wang X, Yuan Y, Li X. Transcranial ultrasound stimulation directly influences the cortical excitability of the motor cortex in parkinsonian mice. Mov Disord. 2020:35(4):693–698. 10.1002/mds.27952. 31829467

[ref291] Wang MB , Boring MJ, Ward MJ, Richardson RM, Ghuman AS. Deep brain stimulation for parkinson's disease induces spontaneous cortical hypersynchrony in extended motor and cognitive networks. Cereb Cortex. 2022:32(20):4480–4491. 10.1093/cercor/bhab496. 35136991 PMC9574237

[ref292] Wang J , Sun J, Gao L, Zhang D, Chen L, Wu T. Common and unique dysconnectivity profiles of dorsal and median raphe in Parkinson's disease. Hum Brain Mapp. 2023:44(3):1070–1078. 10.1002/hbm.26139. 36334274 PMC9875924

[ref293] Waninger S , Berka C, Stevanovic Karic M, Korszen S, Mozley PD, Henchcliffe C, Kang Y, Hesterman J, Mangoubi T, Verma A. Neurophysiological biomarkers of Parkinson's disease. J Parkinsons Dis. 2020:10(2):471–480. 10.3233/JPD-191844. 32116262 PMC7242849

[ref294] Watts RL , Mandir AS. Abnormalities of supplementary motor area (SMA) task related neuronal activity in MPTP parkinsonism. Mov Disord. 1990:5(Suppl. 1):78.2296263

[ref295] Wichmann T . Pathophysiologic basis of movement disorders. Prog Neurol Surg. 2018:33:13–24. 10.1159/000480718. 29332070

[ref296] Wiesman AI , da Silva Castanheira J, Degroot C, Fon EA, Baillet S, Group P-AR, Network QP. Adverse and compensatory neurophysiological slowing in Parkinson's disease. Prog Neurobiol. 2023:231:102538. 10.1016/j.pneurobio.2023.102538. 37832713 PMC10872886

[ref298] Willard AM , Isett BR, Whalen TC, Mastro KJ, Ki CS, Mao X, Gittis AH. State transitions in the substantia nigra reticulata predict the onset of motor deficits in models of progressive dopamine depletion in mice. elife. 2019:8:e42746. 10.7554/eLife.42746. 30839276 PMC6402832

[ref299] Williams SM , Goldman-Rakic PS. Characterization of the dopaminergic innervation of the primate frontal cortex using a dopamine-specific antibody. Cereb Cortex. 1993:3(3):199–222. 10.1093/cercor/3.3.199. 8100725

[ref300] Williams SM , Goldman-Rakic PS. Widespread origin of the primate mesofrontal dopamine system. Cereb Cortex. 1998:8(4):321–345. 10.1093/cercor/8.4.321. 9651129

[ref301] Wu AK , McCairn KW, Zada G, Wu T, Turner RS. Motor cortex stimulation: mild transient benefit in a primate model of Parkinson disease. J Neurosurg. 2007:106(4):695–700. 10.3171/jns.2007.106.4.695. 17432724 PMC4416648

[ref302] Wu T , Long X, Wang L, Hallett M, Zang Y, Li K, Chan P. Functional connectivity of cortical motor areas in the resting state in Parkinson's disease. Hum Brain Mapp. 2011:32(9):1443–1457. 10.1002/hbm.21118. 20740649 PMC6870250

[ref303] Xiang J , Tao Y, Xia Y, Luo S, Zhao Q, Li B, Zhang X, Sun Y, Xia W, Zhang M, et al. Development of an alpha-synuclein positron emission tomography tracer for imaging synucleinopathies. Cell. 2023:186(16):3350–3367.e19. 10.1016/j.cell.2023.06.004. 37421950 PMC10527432

[ref304] Yang X , Song L, Liu Z. The effect of repetitive transcranial magnetic stimulation on a model rat of Parkinson's disease. Neuroreport. 2010:21(4):268–272. 10.1097/WNR.0b013e328335b411. 20087233

[ref305] Yin Z , Zhu G, Liu Y, Zhao B, Liu D, Bai Y, Zhang Q, Shi L, Feng T, Yang A, et al. Cortical phase-amplitude coupling is key to the occurrence and treatment of freezing of gait. Brain. 2022:145(7):2407–2421. 10.1093/brain/awac121. 35441231 PMC9337810

[ref306] Yu H , Sternad D, Corcos DM, Vaillancourt DE. Role of hyperactive cerebellum and motor cortex in Parkinson's disease. NeuroImage. 2007:35(1):222–233. 10.1016/j.neuroimage.2006.11.047. 17223579 PMC1853309

[ref307] Yu Y , Escobar Sanabria D, Wang J, Hendrix CM, Zhang J, Nebeck SD, Amundson AM, Busby ZB, Bauer DL, Johnson MD, et al. Parkinsonism alters Beta burst dynamics across the basal ganglia-motor cortical network. J Neurosci. 2021:41(10):2274–2286. 10.1523/JNEUROSCI.1591-20.2021. 33483430 PMC8018776

[ref308] Zang Z , Song T, Li J, Nie B, Mei S, Zhang C, Wu T, Zhang Y, Lu J. Simultaneous PET/fMRI revealed increased motor area input to subthalamic nucleus in Parkinson’s disease. Cereb Cortex. 2022:33(1):167–175. 10.1093/cercor/bhac059. 35196709

[ref309] Zanjani A , Zakzanis KK, Daskalakis ZJ, Chen R. Repetitive transcranial magnetic stimulation of the primary motor cortex in the treatment of motor signs in Parkinson's disease: a quantitative review of the literature. Mov Disord. 2015:30(6):750–758. 10.1002/mds.26206. 25786995

[ref310] Zhang C , Dou B, Wang J, Xu K, Zhang H, Sami MU, Hu C, Rong Y, Xiao Q, Chen N, et al. Dynamic alterations of spontaneous neural activity in Parkinson's disease: a resting-state fMRI study. Front Neurol. 2019:10:1052. 10.3389/fneur.2019.01052. 31632340 PMC6779791

[ref311] Zhang C , Lai Y, Li J, He N, Liu Y, Li Y, Li H, Wei H, Yan F, Horn A, et al. Subthalamic and Pallidal stimulations in patients with Parkinson's disease: common and dissociable connections. Ann Neurol. 2021:90(4):670–682. 10.1002/ana.26199. 34390280 PMC9292442

[ref312] Zokaei N , Quinn AJ, Hu MT, Husain M, van Ede F, Nobre AC. Reduced cortico-muscular beta coupling in Parkinson's disease predicts motor impairment. Brain Commun. 2021:3(3):fcab179. 10.1093/braincomms/fcab179. 34514395 PMC8421699

